# High-frequency enhanced VaR: A robust univariate realized volatility model for diverse portfolios and market conditions

**DOI:** 10.1371/journal.pone.0303962

**Published:** 2024-05-22

**Authors:** Wei Kuang

**Affiliations:** School of Sport Business, Guangzhou Sport University, Guangzhou, Guangdong, China; JNKVV: Jawaharlal Nehru Krishi Vishwa Vidyalaya, INDIA

## Abstract

In the field of financial risk management, the accuracy of portfolio Value-at-Risk (VaR) forecasts is of critical importance to both practitioners and academics. This study pioneers a comprehensive evaluation of a univariate model that leverages high-frequency intraday data to improve portfolio VaR forecasts, providing a novel contrast to both univariate and multivariate models based on daily data. Existing research has used such high-frequency-based univariate models for index portfolios, it has not adequately studied their robustness for portfolios with diverse risk profiles, particularly under changing market conditions, such as during crises. Our research fills this gap by proposing a refined univariate long-memory realized volatility model that incorporates realized variance and covariance metrics, eliminating the necessity for a parametric covariance matrix. This model captures the long-run dependencies inherent in the volatility process and provides a flexible alternative that can be paired with appropriate return innovation distributions for VaR estimation. Empirical analyses show that our methodology significantly outperforms traditional univariate and multivariate Generalized AutoRegressive Conditional Heteroskedasticity (GARCH) models in terms of forecasting accuracy while maintaining computational simplicity and ease of implementation. In particular, the inclusion of high-frequency data in univariate volatility models not only improves forecasting accuracy but also streamlines the complexity of portfolio risk assessment. This research extends the discourse between academic research and financial practice, highlighting the transformative impact of high-frequency data on risk management strategies within the financial sector.

## Introduction

Value at Risk (VaR) has become one of the most widely used risk measures to control and manage market risk since its adoption by the Basel Committee on Banking Supervision in 1996. VaR is defined as the maximum loss that may be incurred by a trading portfolio, over a given time horizon with a specified probability. Effective VaR forecasts are critical not only for meeting regulatory capital requirements to maintain financial stability but also for making optimal capital allocation and investment decisions to promote financial vitality [1]. There has been a surge in VaR study since the 2008 financial crisis. While large-scale research has focused on developing VaR methodology for a single asset or index, far less attention has been dedicated to exploring its implications for portfolio VaR forecast. This is even though risks are managed on a portfolio basis by financial institutions, with diversification or tail contagion effects being considered. The banks’ minimum market risk capital requirements apply to the consolidated trading books of their financial entities [2].

To forecast the VaR of a portfolio, there are two approaches: 1) fitting a univariate volatility model to the portfolio returns; and 2) using a multivariate model to capture the dynamic covariances or correlations between asset returns. One significant advantage of the univariate model is that it does not require the modelling of asset correlations and other interdependencies. However, the univariate model’s reliance on portfolio weights presents a significant limitation [3]. The weights of the assets within the portfolio change significantly or when the portfolio is rebalanced. These changes can alter the portfolio’s risk profile, and the univariate model may not adequately adjust to these shifts without recalibration, leading to potential inaccuracies in VaR estimation. The question of which model is preferable for portfolio VaR forecasting given portfolio weights has attracted research interest. On the one hand, using a multivariate model to predict the combined dynamics of the assets in the portfolio might lead to better forecasting due to the usage of more information. The additional information, on the other hand, may be jeopardized by the greater uncertainty caused by the large number of parameters to be estimated in the multivariate volatility models [4]. [5] find that higher-dimensional information is irrelevant or even misleading in some situations for portfolio risk management.

[6] find that the benefits of using a multivariate model for forecasting portfolio volatility and VaR are insignificant. [5] suggest that VaR forecast based on the univariate GARCH model is at least as good as the forecasts from the multivariate counterparts within identical innovation distribution families. The complex multivariate models tend to overfit the data [7]. The relative performance of multivariate volatility in VaR forecasts depends more on the distributional assumptions than on the parametric specification of the volatility models [8, 9]. Indeed, a model with skew student *t* innovations outperforms those with symmetric distributions in both univariate and multivariate contexts [10–12]. Moreover, capturing time-varying conditional correlations is also important [13]. Some studies further investigate the value of using Copula functions to allow for a flexible joint distribution for portfolio VaR forecasts [14, 15]. On the other hand, [16] finds mixed evidence about the VaR performance of univariate and multivariate models. [4] show that multivariate models outperform their univariate counterparts to forecast portfolio VaR for relatively large diversified portfolios.

Clearly, there is no conclusive evidence for the superiority of a univariate or multivariate volatility model. In addition, there are some limitations of previous research. First, most studies are based on a portfolio with a few assets, and the findings of any superior multivariate models are difficult to adapt to larger portfolios in practical applications. Second, the reliance on index data or the assumption of equally weighted portfolios in previous studies oversimplifies the intricate nature of individual portfolio risk profiles. Each portfolio’s risk profile is shaped by the specific assets it contains, their respective weights, and the interdependencies among them. By ignoring these nuanced factors, researchers may fail to recognize the significant influence that a portfolio’s unique risk profile has on the selection and performance of the most appropriate risk assessment model. Third, much of the existing analytical work has concentrated on comparing the efficacy of univariate and multivariate GARCH models. This focus has overlooked the potential advantages of integrating high-frequency intraday returns for portfolio VaR forecasting. The inclusion of such detailed frequency data can offer a more refined understanding of market fluctuations, potentially improving the precision of volatility estimates and, by extension, the accuracy of VaR forecasts.

The GARCH models introduced by [17, 18] are based on the daily returns. Despite being unbiased, squares and cross-products of daily returns are inefficient estimators of “integrated” volatility because they only employ one price measurement each period and so contain no information about the price trajectory between measurements. The realized volatility based on the intraday returns gives a more precise and quick-adapting estimate of current volatility than models based on daily data [19–21]. Conditional realized volatility models are shown to outperform GARCH models in volatility forecasting [20–23]. The improvement applies not just to univariate cases, but also multivariate extensions [24–26]. In view of the superior statistical performance, the conditional realized volatility models have been explored in VaR forecasts. [27] were the first to compare the conditional realized volatility model with the GARCH models and find that they deliver equivalent results for VaR forecast. More recent research suggests that realized volatility improves VaR forecasting [28–33]. However, these studies have primarily focused on a single asset or index VaR forecast leaving the significance of intraday data in portfolio VaR forecasting unexplored.

Given the ongoing debate on the efficacy of univariate versus multivariate models in portfolio VaR forecasting and the limitations of existing research, this study aims to address the research question: Can a refined univariate model, enhanced by high-frequency intraday data, provide a more accurate and computationally efficient alternative for portfolio VaR estimation across diverse market conditions and risk profiles? Specifically, we propose extending univariate conditional realized volatility models for portfolio variance estimation, which can then be used for VaR forecasting. The suggested method effectively harnesses realized volatility and correlation information across assets while not explicitly modelling the covariance matrix in a parametric manner. Therefore, it avoids forecasting entire covariance matrices using realized measures, which may suffer from the positive-definite restriction or curve-of-dimensionality problem. This is built on recent studies on utilizing realized volatility for single index VaR forecasting, but we intend to investigate this univariate modelling technique in a portfolio risk management scenario with more granular asset information. The proposed method is motivated by the findings of approximate log-normality and high persistence on realized volatilities [34]. The selection of univariate models draws on insights from [20] which show that for three exchange rates, univariate long-memory models can explain a considerable percentage of the volatility variation, with only modest incremental value from modelling by a vector autoregressive process. The results of this study could be generalized to other assets due to the remarkable similarities in realized volatility across asset and asset classes such as similar unconditional distributions, highly persistent auto-correlation, and strong co-movement [35]. Moreover, the ease of implementation holds promise for high-dimensional applications.

The data include daily returns and realized covariance matrices for a portfolio of ten Down Jones Industrial Average stocks from February 01, 2001 to December 31, 2009, provided by the [24] paper. We investigate the out-of-sample portfolio volatility and VaR forecast of the long memory realized volatility models which include AutoRegressive Fractionally Integrated Moving Average (ARFIMA), heterogeneous autoregressive-realized volatility (HAR-RV) [36], and one of its extension asymmetric HAR-RV [37]. As a benchmark, we report results for the univariate and multivariate GARCH models that explicitly allow for time-varying volatilities and correlations. The evaluation is based on a range of statistical and risk management criteria including VaR exception frequency, loss magnitude, and efficiency. The results show that the realized volatility models produce more informative and accurate portfolio conditional volatility forecasts, resulting in more accurate VaR predictions. The findings are robust against portfolio risk profiles and market conditions.

The remainder of the article is organized as follows. Section Theoretical background provides the theoretical framework for volatility and VaR forecasting. Section Models and methods presents the conditional volatility models and the innovation distributions assumptions. Section Data and Evaluation describes the data, estimation procedure, and evaluation criteria. Section Results and Discussion discuss the empirical results. Section Conclusion concludes.

## Theoretical background

Denote the vector of returns of *n* assets in the portfolio at time *t* by ***R***_*t*_ = (*r*_1,*t*_, …, *r*_*N*,*t*_)′ and portfolio return by $r_{p,t}=W_{t}^{\prime }R_{t}$ where $W_{t}^{\prime }$ is the vector of portfolio weights. Portfolio VaR is defined as the maximum loss that can be experienced over a given time horizon with a certain confidence level *θ*, i.e. $\text{Pr}(r_{p,t}\leq {\text{VaR}}_{p,t}^{\alpha })=\alpha$ where *α* = 1 − *θ* and *r*_*p*,*t*_ is the portfolio’s return over the period. The day ahead portfolio VaR is given by

$$\begin{array}{c} {\text{VaR}}_{p,t}^{\alpha }={\hat{\mu }}_{p,t}+{\hat{\sigma }}_{p,t}{\hat{F}}_{z}^{-1}\left(\alpha \right) \end{array}$$ (1)

where ${\hat{\mu }}_{p,t}$ and ${\hat{\sigma }}_{p,t}$ are the portfolio conditional mean and conditional standard deviation forecasts at time *t*, respectively and ${\hat{F}}_{z}^{-1}$ is the inverse cdf of the standardized returns or innovations, i.e. $\hat{z_{t}}=(r_{p,t}-{\hat{\mu }}_{p,t})/{\hat{\sigma }}_{p,t}$. In order to compute the portfolio VaR, ${\hat{\mu }}_{p,t}$, ${\hat{\sigma }}_{p,t}$ and ${\hat{F}}_{z}^{-1}$ need to be estimated, which can be produced using two alternative conditional sets assessible at time *t* − 1: previous portfolio returns, i.e. $r_{p,t-h}=W_{t-h}^{\prime }R_{t-h}$ or the full vector of past asset returns, i.e. ***R***_*t*−*h*_ = (*r*_1,*t*−*h*_, …, *r*_*n*,*t*−*h*_)′. The former instance results in a univariate model for portfolio returns, while the latter results in a multivariate model [4].

Although conditional mean returns are difficult to forecast due to weak dependence, conditional volatility is both time-varying and highly predictable. Empirical research on conditional volatility modelling has exploded since the GARCH model was introduced by [17, 18] to capture the volatility clustering effect. See [38–42], and among others for a survey of ARCH models including the multivariate extensions. The finding of time variation in conditional covariance and correlations leads to the development of multivariate GARCH models. For portfolio VaR forecast, conditional volatility of portfolio returns can be estimated by either: (1) fitting a univariate GARCH model to the portfolio returns; (2) using a multivariate GARCH volatility model to forecast the conditional variance of each asset in the portfolio, as well as the conditional correlations between assets returns [16].

Consider an *N* × 1 vector of continuous logarithmic prices, ***p***(*t*) which follows the *N*-dimensional continuous-time diffusion,

$$\begin{array}{c} dp(t)=M(t)dt+\Omega (t)dW(t),\;t\in [0,T] \end{array}$$ (2)

where ***M***(*t*) is an *N* × 1 instantaneous drift, Ω(*t*) denotes the *N* × *N* instantaneous diffusion matrix, and ***W***(*t*) is an *N* × 1 dimensional vector of independent standard Brownian motions. The stochastic process governing the discretely observed *N* × 1 daily logarithmic return vector, ***R***_*t*_ = ***p***(*t*) − ***p***(*t* − 1), is given by

$$\begin{array}{c} R_{t}=M_{t}+z_{t}\Omega_{t} \end{array}$$ (3)

where ***z***_*t*_ is an *N* × 1 vector of standard normally distributed, serially uncorrelated random variables and Ω_*t*_ is an *N* × *N* integrated covariance matrix defined as

$$\begin{array}{c} \Omega_{t}=\int_{t-1}^{t}\Omega_{s}\;ds \end{array}$$ (4)

The integrated covariance matrix given by Eq (4) is unobservable. An unbiased estimator of Ω_*t*_ is given by the squared daily returns

$$\begin{array}{c} \Omega_{t}=R_{t}R_{t}^{\prime } \end{array}$$ (5)

The estimator of the integrated covariance matrix based on daily returns forms the basis for a wide range of interdaily conditional volatility models such as the GARCH models. Despite being unbiased, the estimator using squared daily returns is inefficient because the signal that it contains about Ω_*t*_ is dominated by noise [43]. [44] show that realized covariance, the sum of intraday cross products of returns, converges in probability to the quadratic covariation under mild regularity conditions. Let ***R***(*t*, Δ) denote the *n* × 1 vector of logarithmic returns over the [*t* − Δ, *t*] time interval, ***R***(*t*, Δ) = ***p***(*t*) − ***p***(*t* − Δ). The *n* × *n* realized covariance matrix for the unit time interval [*t* − 1, *t*] can be defined as

$$\begin{array}{c} \Sigma_{t,\Delta }=\sum_{j=1}^{1/\Delta }R(t-1+j\Delta ,\Delta )R{\left(t-1+j\Delta ,\Delta \right)}^{\prime } \end{array}$$ (6)

where Σ_*t*,Δ_ will converge to the integrated covariance matrix Ω_*t*_ for finer and finer sampled high-frequency returns. Σ_*t*,Δ_ generally satisfies the positive definiteness condition as long as *n* < 1/Δ [20]. The accuracy of this approach, however, is limited by the market microstructure effects which distort the measurement of returns at high frequencies, making measured returns no longer satisfy the regularity conditions required for the consistency properties of realized volatility. Returns measured at intervals of five to thirty minutes are shown to provide the optimal balance of precision and market microstructure frictions [20, 34].

[19] show that the realized variance and covariance of stock returns are approximately log-normally distributed and highly persistent, which are well represented by long-memory processes. This implies that the realized volatility of a portfolio which is a linear combination of asset returns may have a similar attribute, namely approximating a log-normal distribution with a long memory. [20] find similar results for currencies and their cross rate, and suggest using a fractionally integrated model to forecast each element of the realized covariance matrix or its Cholesky decomposition. This is because the continuously compounded return on the cross rate should be equal to the difference between the two pairs of currency returns in the absence of triangle arbitrage.

Rather than forecasting Σ_*t*,Δ_, this paper proposes to extend long memory realized volatility models to forecast portfolio variance, which can directly feed into VaR forecasts. Specifically, given the vector of portfolio weights ***W***_*t*_, the portfolio realized variance can be specified as

$$\begin{array}{c} RV_{p.t}=W_{t}^{\prime }\Sigma_{t,\Delta }W_{t} \end{array}$$ (7)

where *RV*_*p*.*t*_ is a linear combination of individual elements of Σ_*t*,Δ_. For example, the realized variance of a bivariate portfolio can be calculated as

$$\begin{array}{c} \begin{array}{cc} RV_{B,t} & =\left[w_{1,t},w_{2,t}\right][\begin{array}{c} \sigma_{11,t}\;\sigma_{12,t}\\ \sigma_{21,t}\;\sigma_{22,t} \end{array}][\begin{array}{c} w_{1,t}\\ w_{2,t} \end{array}]\\ & =w_{1,t}^{2}\sigma_{11,t}+2\times w_{1,t}w_{2,t}\sigma_{12,t}+w_{2,t}^{2}\sigma_{22,t} \end{array} \end{array}$$ (8)

where *w*_*i*,*t*_ is the weight for asset *i* at time *t*; *σ*_*ij*,*t*_ is the realized variance of an individual asset for (*i* = *j*) and realized covariance between assets for (*i* ≠ *j*). This is essentially the realized volatility based on the aggregated returns of all the assets in the portfolios. The proposed method takes into account: 1) the complexity of forecasting realized covariance matrix Σ_*t*,Δ_ with multivariate modelling constraint; and 2) the long-run dependency of realized volatility with univariate modelling flexibility.

There are three challenges to incorporating realized measures into multivariate volatility models for tail risk forecasting. First, the conditional realized volatility models must be parameterized in such a way that the forecasted covariance matrices are positive definite in high-dimensional settings. Second, the multivariate volatility models, particularly those based on the GARCH specification have a large number of parameters, resulting in the curse-of-dimensionality problem. Third, the conditional distributions of innovations are analytically tractable only when the return distribution is closed under linear transformation, i.e. the linear combinations of *r*_*i*,*t*_ have the same distribution as the marginal distribution of returns *r*_*i*,*t*_ [3]. The proposed method, however, shares the benefit of univariate modelling in that it is parsimonious and allows for flexible distribution to capture skewness and excess kurtosis in the innovation process without adding significant computational costs. The details of the models are discussed in the following section.

## Models and methods

The conditional mean return is assumed to be constant. To forecast portfolio variance, three intradaily volatility models are considered, including the ARFIMA model suggested by [19, 20] for realized volatility modelling, the HAR-RV model [36] based on the Heterogeneous Market Hypothesis of [45], and one of its extension asymmetric HAR-RV model [37] to allow for leverage effect. The conventional HAR-RV model is well known for capturing the long memory features observed in financial time series data [36]. Furthermore, the inclusion of asymmetry extensions in the HAR-RV model is consistent with empirical evidence suggesting that volatility is frequently subject to the leverage effect, in which negative market movements generate larger changes in volatility than positive market movements of the same magnitude. While alternative models, such as those incorporating jumps certainly offer additional lenses through which to analyze volatility, our objective was to strike a balance between model complexity and interpretability. The model description is provided in section. Some representative GARCH-type models are included for comparison. The univariate GARCH models include the standard GARCH [18] to capture volatility clustering, as well as various extensions to address leverage effects such as the Glosten-Jagannathan-Runkle (GJR)GARCH [46] and the Asymmetric Power Autoregressive Conditional Heteroskedasticity (APARCH) [47], and long memory property such as the Fractionally Integrated Generalized Autoregressive Conditional Heteroskedasticity (FIGARCH) [48]. The multivariate GARCH models include the RiskMetrics Exponentially Weighted Moving Average (EWMA) [49] and the Dynamic Conditional Correlation (DCC) model [50]. Because these GARCH models are standard, their specifications are presented in Table 1 for reference.

**Table 1:** GARCH model specification.

Model	Specifcation	Innovation
**Panel A: Univariate**		
GARCH	$\sigma_{p,t}^{2}=\omega +\alpha \varepsilon_{t-1}^{2}+\beta \sigma_{p,t-1}^{2}$	FHS,N,ST
GJRGARCH	$\sigma_{p,t}^{2}=\omega +\alpha \varepsilon_{t-1}^{2}+\beta \sigma_{p,t-1}^{2}+\gamma I(\varepsilon_{t-1}<0)$	N,ST
APARCH	$\sigma_{p,t}^{2}={\left[\omega +\alpha {\left(|\varepsilon_{t-1}|-\gamma \varepsilon_{t-1}\right)}^{\delta }+\beta \sigma_{p,t-1}^{\delta }\right]}^{1/\delta }$	N,ST
FIGARCH	$\sigma_{p,t}^{2}=\frac{\omega }{1-\beta (L)}+[1-\frac{\left(1-\alpha \left(L\right)\right){\left(1-L\right)}^{d}}{1-\beta (L)}]\varepsilon_{t}^{2}$	N,ST
**Panel B: Multivariate**		
EWMA	${\hat{\Sigma }}_{t}^{ewma}=\lambda {\hat{\Sigma }}_{t-1}^{ewma}+\left(1-\lambda \right)R_{t}R_{t}^{\prime }$	MVN
DCC	${\hat{\Sigma }}_{t}^{DCC}=D_{t}C_{t}D_{t}$ $D_{t}=\text{diag}(h_{11,t}^{1/2}\ldots h_{nn,t}^{1/2})$ where *h*_*ii*,*t*_ is defined as a GARCH(1,1) model *C*_*t*_ = (diag*Q*_*t*_)^−1/2^*Q*_*t*_(diag*Q*_*t*_)^−1/2^ $Q_{t}=\left(1-\alpha -\beta \right)\bar{Q}+\alpha u_{t-1}u_{t-1}+\beta Q_{t-1}$ where $u_{i,t}=\varepsilon_{i,t}/\sqrt{h_{ii,t}}\;\left(i=1,\ldots ,n\right)$ $\bar{Q}$ is the *n* × *n* unconditional variance matrix of *u*_*t*_	MVT

**Notes:** The table shows the specification of univariate and multivariate GARCH models with the selected innovation distributions for VaR forecasts. The innovation distributions include filtered historical simulation (FHS), normal distribution (N) and skewed student distribution for the univariate GARCH models, and the multivariate normal (MVN) for EWMA and multivariate Student *t* (MVT) distribution for the DCC model.

Two innovation distributions are considered in univariate models: the standard normal and the skewed student distribution [51] that addresses the skewness and excess kurtosis commonly observed for financial return data [10, 52, 53]. We also include the filter historical simulation (FHS) approach [54] which combines the standard GARCH with the empirical distribution of innovation for VaR forecast. For the multivariate models, we follow [4] to consider two alternative multivariate distributions: the multivariate normal distribution for the EWMA model and the multivariate student *t* distribution for the DCC model. We intend to keep the innovation distribution tractable so that it can be applied to higher-dimensional settings without raising estimation issues or computation costs. Some more sophisticated distributions such as the multivariate skewed Student *t* distribution of [11] do not have an analytical formula for computing VaR using conditional volatilities which require Monte-Carlo simulation. The details of the innovation distributions are covered in section.

### Volatility models

#### ARFIMA model

The long memory property of realized volatility is captured by the fractionally differencing parameter *d*.

$$\begin{array}{c} \left(1-\psi \left(L\right)\right){\left(1-L\right)}^{d}(lrv_{t}^{\left(d\right)}-\mu )=\left(1+\delta \left(L\right)\right)u_{t} \end{array}$$ (9)

where $lrv_{t}^{\left(d\right)}=log\left(RV_{p,t}\right)$ is the logarithm of the daily portfolio realized variance defined in Eq (7).

#### HAR-RV model

The persistence of realized volatility is approximated by an autoregressive structure using daily, weekly, and monthly volatility components.

$$\begin{array}{c} lrv_{t}^{\left(d\right)}=\alpha_{0}+\alpha_{d}lrv_{t-1}^{\left(d\right)}+\alpha_{w}lrv_{t-1}^{\left(w\right)}+\alpha_{m}lrv_{t-1}^{\left(m\right)}+\mu_{t} \end{array}$$ (10)

where $lrv_{t}^{\left(d\right)}=log\left(RV_{p,t}\right)$ is the logarithm of the daily portfolio realized variance defined in Eq (7); $lrv_{t}^{\left(h\right)}=(1/h)(lrv_{t}+lrv_{t-1}+lrv_{t-2}+\ldots +lrv_{t-h})$ with *h* = *w* = 5 and *h* = *m* = 22 being the weekly and monthly components respectively.

#### Asymmetric HAR-RV model

The model contains additional lagged (absolute) standardized returns occurring at different time horizons: daily, weekly, and monthly.

$$\begin{array}{c} \begin{array}{cc} lrv_{t}^{\left(d\right)} & =\alpha_{0}+\alpha_{d}lrv_{t-1}^{\left(d\right)}+\alpha_{w}lrv_{t-1}^{\left(w\right)}+\alpha_{m}lrv_{t-1}^{\left(m\right)}+\vartheta_{d}z_{t-1}^{\left(d\right)}+\\ & \vartheta_{w}z_{t-1}^{\left(w\right)}+\vartheta_{m}z_{t-1}^{\left(m\right)}+\gamma_{d}|z_{t-1}^{\left(d\right)}|\gamma_{w}|z_{t-1}^{\left(w\right)}|+\gamma_{m}|z_{t-1}^{\left(m\right)}|+\mu_{t} \end{array} \end{array}$$ (11)

where $z_{t}^{\left(h\right)}=\sum_{i=1}^{h}r_{p,t-i+1}/\sqrt{\sum_{i=1}^{h}RV_{p,t-i+1}}$ are the daily (*d*), weekly (*w*), and monthly (*m*) standardized returns.

The heteroscedasticity in the residuals is expected to persist due to the variance of the realized volatility estimator [55]. Therefore, the GARCH(1,1) with a normally distributed innovation is employed to account for the conditional heteroscedasticity of the residuals in the conditional realized volatility models.

$$\begin{array}{c} u_{t}=\sigma_{u,t}\varepsilon_{t},\;\varepsilon_{t}∼N\left(0,1\right) \end{array}$$ (12)

$$\begin{array}{c} \sigma_{u,t}^{2}=\omega_{u}+\alpha_{u}u_{t-1}^{2}+\beta_{u}\sigma_{u,t-1}^{2} \end{array}$$ (13)

By definition $\text{exp}(u_{t})∼\text{log}N(0,\sigma_{u}^{2})$ when $u_{t}∼N(0,\sigma_{u}^{2})$. Therefore, the conditional realized volatility can be computed as

$$\begin{array}{c} RV_{t}=\text{exp}\left(lrv_{t}-{\hat{u}}_{t}+0.5{\hat{\sigma }}_{u^{t}}^{2}\right). \end{array}$$ (14)

where ${\hat{u}}_{t}$ denotes the estimated value of *u*_*t*_ by the respective realized volatility models and ${\hat{\sigma }}_{u^{t}}^{2}$ is the estimated variance defined in Eq (13).

### Innovation distribution

#### Normal distribution

If the innovation follows a standard normal distribution, i.e. *z*_*t*_ ∼ *i*.*i*.*dN*(0, 1), there is no additional parameter to be estimated. The day-ahead VaR forecast is given by

$$\begin{array}{c} {\text{VaR}}_{p,t}^{\alpha }={\hat{\mu }}_{p,t}+{\hat{\sigma }}_{p,t}\Phi^{-1}\left(\alpha \right) \end{array}$$ (15)

where *Φ*^−1^(*α*) is the *α* quantile of the standard normal distribution.

#### Skewed student distribution

The quantile function is derived by [56] as follows

$$\begin{array}{c} c_{\alpha ,\nu ,\xi }^{skst}=\{\begin{array}{cc} \{\frac{1}{\xi }c_{\alpha ,\nu }^{st}[\frac{\alpha }{2}(1+\xi^{2})]-m\}/s, & \text{if}\;\alpha <\frac{1}{1+\xi^{2}}\\ \{-\xi c_{\alpha ,\nu }^{st}[\frac{1-\alpha }{2}(1+\xi^{-2})]-m\}/s, & \text{if}\;\alpha \geq \frac{1}{1+\xi^{2}} \end{array} \end{array}$$ (16)

where $c_{\alpha ,\nu ,\xi }^{skst}$ is the *α*th quantile of the unit variance skewed student distribution with *ν* > 2 degrees of freedom and asymmetric parameter *ξ* > 0; $c_{\alpha ,\nu }^{st}$ denotes the quantile function of the standardized Student-*t* density function; $m=\frac{\Gamma (\frac{\nu +1}{2})\sqrt{\nu -2}}{\sqrt{\pi }\Gamma (\frac{\nu }{2})}(\xi -\frac{1}{\xi })$ and $s=\sqrt{(\xi^{2}+\frac{1}{\xi^{2}}-1)-m^{2}}$ are the mean and standard deviation of the non-standardized skewed student distribution, respectively. The day-ahead VaR forecast is then given by

$$\begin{array}{c} {\text{VaR}}_{p,t}^{\alpha }={\hat{\mu }}_{p,t}+{\hat{\sigma }}_{p,t}c_{\alpha ,\nu ,\xi }^{skst} \end{array}$$ (17)

#### Filtered historical simulation

The filtered historical simulation (FHS) introduced by [54] makes no distribution assumptions regarding the innovation. It employs innovation to develop hypothetical future possibilities, which can be accomplished by resampling or bootstrapping. We use the empirical distribution function (EDF) with the historical cumulative distribution of standardized returns in this paper. As a result, the *α* quantile is based on the empirical data presented below.

$$\begin{array}{c} G_{\alpha }^{-1}=\text{Quantile}\left(\alpha \right)\left\{{\left\{z_{i}\right\}}_{i=1}^{t}\right\} \end{array}$$ (18)

The day-ahead VaR forecast is then given by

$$\begin{array}{c} {\text{VaR}}_{p,t}^{\alpha }={\hat{\mu }}_{p,t}+{\hat{\sigma }}_{p,t}G_{\alpha }^{-1} \end{array}$$ (19)

#### Multivariate distribution

The portfolio conditional variance is denoted by ${\hat{\sigma }}_{p,t}=W_{t}^{\prime }\hat{\Sigma_{t}}W_{t}$ where $\hat{\Sigma_{t}}$ is the conditional covariance matrix forecast. The day-ahead VaR forecast under the multivariate normal distribution assumption is given by

$$\begin{array}{c} {\text{VaR}}_{p,t}^{\alpha }={\hat{\mu }}_{p,t}+{\hat{\sigma }}_{p,t}z_{\alpha } \end{array}$$ (20)

where *z*_*α*_ is the *α* quantile of the normal distribution. Under the assumption of multivariate Student *t* distribution, the *z*_*α*_ is replaced by *t*_*α*,*ν*_ where *ν* is the degrees of freedom.

## Data and evaluation

### Data and estimation

To build an equity portfolio, we use the data of 10 Dow Jones Industrial Average (DJIA) stocks from the [24] paper. The data are available from the website of Oxford Man Institute of Quantitative Finance. The underlying high-frequency data came from the Trade and Quote database of the New York Stock Exchange. The realized covariance matrix was constructed using intraday returns sampled at a 10-minute frequency. The data set includes open-to-close and close-to-close daily returns, as well as the vech of the 10 × 10 realized covariance matrix for the following stocks: Bank of America (BAC), JP Morgan (JPM), International Business Machines (IBM), Microsoft (MSFT), Exxon Mobil (XOM), Alcoa (AA), American Express (AXP), Du Pont (DD), General Electric(GE) and Coca Cola (KO) from February 01, 2001 to December 31, 2009 for a total of 2,242 observations. We use open-to-close daily returns for all interdaily, i.e. GARCH models, given that the realized covariance matrix does not account for overnight returns. The sample is divided into an initial estimation period of 1,000 observations from February 01, 2001 to January 26, 2005 and 1,242 out-of-sample observations from January 27, 2005 to December 31, 2009. The out-of-sample period covers calm and turbulent times, including the 2008 financial crisis. The models are initialized using the estimating period and the volatility and VaR forecasts are created for Day 1,001. After that, the estimation window is then advanced by one day. The models are re-evaluated and used to generate forecasts for Day 1,002, and so on until the sample is completed. The key advantage of using a rolling window of 1000 observations is that it combines the most recent market data while discarding out-of-date observations.

**Fig 1:**
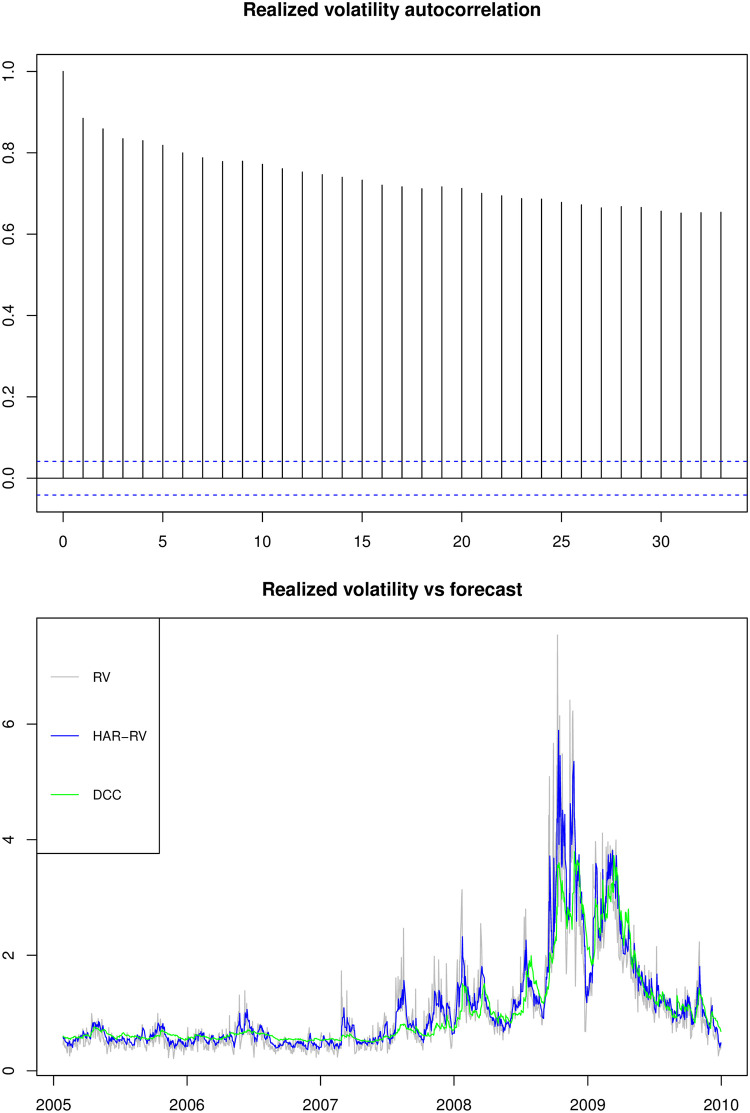
Realized volatility. **Notes:** The upper graph shows the autocorrelation of portfolio logarithm realized standard deviation over the full sample period from February 01, 2001 to December 31, 2009 (2,242 observations). The lower graph shows the portfolio realized standard deviation against forecasts from HAR-RV and DCC models over the out-of-sample period from January 27, 2005 to December 31, 2009 (1,242 observations).

Table 2 reports the summary statistics for each return series as well as the equally weighted portfolio return and its logarithmic realized standard deviation. The mean returns on individual stocks and the portfolio are close to zero. All returns show skewness and excess kurtosis with the normality assumption being rejected in all cases. The ARCH-LM test of [17] for up to twentieth-order serial correlation in squared returns reveals evidence of considerable volatility clustering. The decision to consider autocorrelation and conditional heteroskedasticity of order up to 20 was based on a comprehensive review of the literature [27, 57, 58], which allows for the detection of both short-term and moderately long-term dependencies in the data. The logarithmic realized standard deviation (LRV), on the other hand, follows more closely to the normal distribution but has a strong serial correlation and ARCH effect. Moreover, it should be noted that the Ljung-Box statistics of LRV is an order of magnitude larger than the ARCH-LM statistics of daily squared returns. This could be explained by the fact that daily squared returns are relatively noisy volatility proxies when compared to the daily realized volatilities [43]. The noise masks the significant persistence in the underlying volatility dynamics [20]. The upper panel of Fig 1 provides graphical illustration of the sample autocorrelation of the portfolio realized logarithmic standard deviation out to over 30 days. The autocorrelation is significantly above the 95% confidence band and decays slowly, indicating a long-memory mechanism. The lower panel compares the one-day ahead volatility forecast of HAR-RV model and the DCC model against the realized volatility over the out-of-sample period. Realized portfolio volatility fluctuates dramatically over time. The HAR-RV model generates volatility forecasts that respond to changes in the underlying unobserved volatility levels faster than the DCC model, although the DCC model captures the volatility and correlation dynamics using stock daily returns.

**Table 2:** Summary statistics.

	**BAC**	**JPM**	**IBM**	**MSFT**	**XOM**	**AA**
Mean	0.000	0.000	0.000	0.000	0.000	0.000
Stdev	2.390	2.198	1.300	1.436	1.291	2.108
Skew	0.328	0.577	0.013	0.247	-0.191	-0.681
Kurt	18.791	14.032	3.313	3.150	8.629	6.889
B-J	33095.445	18558.477	1028.657	953.072	6986.231	4618.695
*p*-val	0.000	0.000	0.000	0.000	0.000	0.000
L-B	65.973	55.947	31.399	50.741	30.240	33.217
*p*-val	0.000	0.000	0.050	0.000	0.066	0.032
LM	678.506	349.137	466.498	448.478	542.226	464.919
*p*-val	0.000	0.000	0.000	0.000	0.000	0.000
	**AXP**	**DD**	**GE**	**KO**	**EW**	**LRV**
Mean	0.000	0.000	0.000	0.000	0.000	-0.191
Stdev	2.085	1.452	1.636	1.064	1.274	0.579
Skew	0.316	0.030	0.217	0.105	-0.069	0.603
Kurt	8.220	4.277	7.952	3.912	8.680	0.196
B-J	6364.417	1714.164	5939.919	1438.508	7057.671	139.617
*p*-val	0.000	0.000	0.000	0.000	0.000	0.000
L-B	40.735	17.514	62.830	70.741	35.342	27020.677
*p*-val	0.004	0.619	0.000	0.000	0.018	0.000
LM	614.165	462.563	473.642	429.903	546.499	1239.100
*p*-val	0.000	0.000	0.000	0.000	0.000	0.000

**Notes:** The table reports the mean, standard deviation (Stdev), skewness (Skew), excess kurtosis (Kurt), Jarque-Bera (B-J) test for the daily returns in percentage of 10 DJIA stocks including Bank of America (BAC), JP Morgan (JPM), International Business Machines (IBM), Microsoft (MSFT), Exxon Mobil (XOM), Alcoa (AA), American Express (AXP), Du Pont (DD), General Electric (GE) and Coca Cola (KO) as well as the equally-weighted 10 DJIA stocks portfolio returns (EW) and its logarithmic realized standard deviation (LRV) over the full sample period February 01, 2001 to December 31, 2009 (2,242 observations). The statistics and *p*-values of the Jarque-Bera (B-J) normality test, the Ljung-Box (L-B) test for autocorrelation of order up to 20, and the ARCH-LM test for autoregressive conditional heteroskedasticity of order up to 20 are also reported.

The estimation results of the univariate ARMA(0,0)-GARCH models using daily returns and the ARFIMA and HAR-RV type models using intraday data are presented in Tables 3 and 4, respectively. The estimation is based on the entire sample period for illustration. We first examine Table 3. The conditional mean returns *μ* are not statistically significantly different from zero in all the models. The parameter *β* of all GARCH models is close to 1, indicating the persistence of volatility. The FIGARCH model’s fractional differencing parameter *d* shows a moderate degree of long memory. The GJRGARCH model’s leverage parameter *γ* is positive and significant, suggesting a leverage effect. Moreover, it should be noted that GARCH filtering removes the time-series dynamics from the return series but not all of the excess kurtosis. The estimates for the skewed student distribution parameters (*ξ* and *ν*) indicate negative skewness and fat tails. We then proceed to Table 4. The fractional differencing parameter *d* in the ARFIMA model, as well as the highly significant and positive *α*_*d*_, *α*_*w*_, and *α*_*m*_ in the HAR-RV and the Asym.HAR-RV model demonstrates the persistence of the realized volatility series. The leverage parameters *ϑ*_(*d*)_, *ϑ*_(*w*)_, *ϑ*_(*m*)_ are all negative and statistically significant, indicating that daily, weekly and monthly shocks all increase volatility. Positive and statistically significant estimates for *γ*_(*d*)_, *γ*_(*w*)_, and *γ*_(*m*)_ suggest heterogeneous size effects. The insignificant ARCH-LM statistics show that the GARCH(1,1) process removes the “volatility of volatility” clustering. Furthermore, the diagnostic statistics of standardized return residuals indicate volatility dynamics have been reduced to a substantial extent with minimal autocorrelation. However, the standard residuals still exhibit significant non-normality.

**Table 3:** GARCH models estimation.

GARCH		GJRGARCH		FIGARCH		APARCH	
Coef	stats	Coef	stats	Coef	stats	Coef	stats
**Panel A: Estimation results**							
*μ*	0.000	*μ*	0.000	*μ*	0.000	*μ*	0.000
	0.999		0.999		0.999		0.999
*ω*	0.005	*ω*	0.004	*ω*	0.021	*ω*	0.005
	0.028		0.010		0.006		0.000
*α*	0.072	*α*	0.000	*α*	0.000	*α*	0.044
	0.000		1.000		1.000		0.000
*β*	0.926	*β*	0.943	*β*	0.575	*β*	0.947
	0.000		0.000		0.000		0.000
*ξ*	0.934	*γ*	0.111	*d*	0.575	*γ*	0.998
	0.000		0.000		0.000		0.000
*ν*	7.983	*ξ*	0.926	*ξ*	0.929	*δ*	1.392
	0.000		0.000		0.000		0.000
		*ν*	9.446	*ν*	8.114	*ξ*	0.926
			0.000		0.000		0.000
						*ν*	9.694
							0.000
**Panel B: Standardized residuals**							
B-J	104.197	B-J	83.778	B-J	88.596	B-J	76.448
	0.000		0.000		0.000		0.000
L-B	23.666	L-B	23.661	L-B	23.894	L-B	23.351
	0.257		0.258		0.247		0.272
LM	18.352	LM	20.581	LM	10.574	LM	22.975
	0.564		0.422		0.957		0.290

**Notes:** Panel A reports the parameters and *p*-values underneath based on the robust standard errors of GARCH-type models with SkewedT innovations over the full sample period from February 01, 2001 to December 31, 2009 (2,242 observations). Panel B reports the diagnostic statistics and the p-values underneath of the standardized residuals, including the Jarque-Bera normality test (B-J), the Ljung-Box test (L-B) for autocorrelation of order up to 20, and the ARCH-LM test for autoregressive conditional heteroskedasticity of order up to 20.

**Table 4:** ARFIMA and HAR-RV models estimation.

GARCH		GJRGARCH		FIGARCH		APARCH	
Coef	stats	Coef	stats	Coef	stats	Coef	stats
**Panel A: Estimation results**							
*μ*	-0.379	*α*_0_	-0.022	*α*_0_	-0.119	*γ*_*d*_	0.062
	0.136		0.054		0.000		0.000
*d*	0.500	*α*_*d*_	0.341	*α*_*d*_	0.286	*γ*_*w*_	0.093
	0.000		0.000		0.000		0.033
*ω*_*μ*_	0.008	*α*_*w*_	0.482	*α*_*w*_	0.470	*γ*_*m*_	0.135
	0.033		0.000		0.000		0.114
*α*_*μ*_	0.038	*α*_*m*_	0.145	*α*_*m*_	0.210	*ω*_*μ*_	0.006
	0.003		0.000		0.000		0.069
*β*_*μ*_	0.929	*ω*_*μ*_	0.007	*ϑ*_*d*_	-0.054	*α*_*μ*_	0.035
	0.000		0.022		0.000		0.008
LM	13.350	*α*_*μ*_	0.039	*ϑ*_*w*_	-0.119	*β*_*μ*_	0.937
	0.862		0.001		0.000		0.000
		*β*_*μ*_	0.930	*ϑ*_*m*_	-0.130	LM	18.810
			0.000		0.030		0.534
		LM	18.266				
			0.570				
**Panel B: Standardized residuals**							
B-J	52.771	B-J	59.722	B-J	58.818		
	0.000		0.000		0.000		
L-B	22.764	L-B	20.877	L-B	20.999		
	0.301		0.404		0.397		
LM	28.473	LM	28.045	LM	38.068		
	0.099		0.108		0.009		
*ξ*	0.927	*ξ*	0.927	*ξ*	0.922		
*ν*	11.912	*ν*	11.497	*ν*	11.381		

**Notes:** Panel A reports the parameters and *p*-values underneath based on the robust standard errors of ARFIMA-GARCH, HAR-RV-GARCH and ASY-HAR-RV-GARCH models over the full sample period from February 01, 2001 to December 31, 2009 (2,242 observations). Panel B shows the skewed student parameters and the diagnostic statistics with the p-values underneath of the standardized residuals, including the Jarque-Bera normality test (B-J), the Ljung-Box test (L-B) for autocorrelation of order up to 20, and the ARCH-LM test for autoregressive conditional heteroskedasticity of order up to 20.

### Forecast evaluation

The relative performances of the models are assessed using both statistical and economic criteria. Although there are many alternative backtesting approaches available, we focus on a sample selection of classic backtesting processes that should provide sufficient evidence for the paper’s results. The statistical criteria examine the accuracy of portfolio volatility forecasting using Root Mean Square Error (RMSE) and Mean Absolute Error (MAE) metrics, as well as the efficiency and information contents using the Mincer-Zarnowitz regression. The portfolio realized volatility $\sigma_{p,t}=\sqrt{RV_{p,t}}$ serves as the evaluation benchmark. The risk management criteria examine the VaR forecast performance in terms of exception frequency (unconditional and conditional coverage tests), exception magnitude (Berkowitz test), joint frequency and magnitude (tick loss and quadratic loss), and VaR efficiency (based on mean and standard deviations).

#### Root Mean Square Error (RMSE)

$$\begin{array}{c} \text{RMSE}=\sqrt{\frac{1}{T}\sum_{t=1}^{T}{\left({\hat{\sigma }}_{p,t}-\sigma_{p,t}\right)}^{2}} \end{array}$$ (21)

#### Mean Absolute Error (MAE)


$$\begin{array}{c} \text{MAE}=\frac{1}{T}\sum_{t=1}^{T}|{\hat{\sigma }}_{p,t}-\sigma_{p,t}| \end{array}$$ (22)

#### Mincer-Zarnowitz (MZ) Regression


$$\begin{array}{c} \sigma_{p,t}=\alpha +\beta {\hat{\sigma }}_{p,t}+\varepsilon_{t} \end{array}$$ (23)

A forecast is conditionally unbiased (i.e., weak-form efficient) if and only if *α* = 0 and *β* = 1 in the MZ regression. The *R*-squared coefficient indicates the explanatory power of each of the model’s forecasts, irrespective of any bias or inefficiency.

#### Unconditional coverage

The unconditional coverage test of [59] examines the null hypothesis that the exception rate is statistically equal to the expected value given the confidence level. The test statistic follows an asymptotic *χ*^2^ distribution with one degree of freedom

$$\begin{array}{c} {\text{LR}}_{uc}^{\alpha }=-2\text{ln}[\frac{\alpha^{n_{1}}{\left(1-\alpha \right)}^{n_{0}}}{{\hat{\pi }}^{n_{1}}{\left(1-\hat{\pi }\right)}^{n_{0}}}]∼\chi_{1}^{2} \end{array}$$ (24)

where *n*_1_ represents the number of exceptions, *n*_0_ represents the number of non-exception, $\hat{\pi }=n_{1}/\left(n_{0}+n_{1}\right)$ is the estimated proportion of exceptions, and *α* = 1 − *p* is the probability of an exception for a given confidence level *p*.

#### Conditional coverage

The conditional coverage test *LR*_*cc*_ of [60] jointly examines whether total exceptions are equal to the expected and whether exceptions are distributed independently. The statistic follows an asymptotic *χ*^2^ distribution with two degrees of freedom.

$$\begin{array}{c} {\text{LR}}_{cc}^{\alpha }=-2\text{ln}[\frac{{\left(1-\alpha \right)}^{n_{0}}\alpha^{n_{1}}}{{\left(1-\pi_{01}\right)}^{n_{00}}\pi_{01}^{n_{01}}{\left(1-\pi_{11}\right)}^{n_{10}}\pi_{11}^{n_{11}}}]∼\chi_{2}^{2} \end{array}$$ (25)

where *π*_*ij*_ denotes the number of times that state *j* follows state *i* for *i*, *j* = 0, 1 and $\pi_{i,j}=\frac{n_{ij}}{\sum_{j}n_{ij}}$; state 0 denotes that no exception of VaR forecast occurs and state 1 denotes that exception occurs.

#### Berkowitz test

The tail test of [61] examines whether the magnitude of observed VaR exceptions is consistent with the underlying VaR model. Suppose *r*_*p*,*t*_ is the ex-pose portfolio returns and $\hat{f}\left(\cdot \right)$ is the ex-ante forecasted loss density with cdf of $\hat{F}\left(\cdot \right)$. $\hat{F}\left(r_{p,t}\right)$ is *i*.*i*.*d* and distributed uniformly on (0, 1) according to [62]. The empirical quantile series can be transformed into a standard normal series using the inverse Normal cdf, i.e. $z_{t}=\Phi^{-1}(\hat{F}\left(r_{p,t}\right))$. Then *z*_*t*_ is treated as a censored normal random variable with the censoring tied to the target confidence level of the VaR estimates. Specifically, *z*_*t*_ is transformed into $z_{t}^{*}$ as follows

$$\begin{array}{c} z_{t}^{*}=\{\begin{array}{cc} {\text{VaR}}_{p,t}^{\alpha }, & \text{if}\;z_{t}\geq {\text{VaR}}_{p,t}^{\alpha }\\ z_{t}, & \text{if}\;z_{t}<{\text{VaR}}_{p,t}^{\alpha } \end{array} \end{array}$$ (26)

If the empirical quantiles are generated correctly by the VaR model, the *z*_*t*_ series should be identically distributed with zero unconditional mean *μ* and unit unconditional standard deviation *σ*. The log-likelihood function is used to estimate *μ* and *σ* jointly

$$\begin{array}{c} L^{\alpha }(\mu ,\sigma |z_{t}^{*})=\sum_{z_{t}^{*}<{\text{VaR}}_{p,t}^{\alpha }}\text{ln}\frac{1}{\sigma }\phi (\frac{z_{t}-\mu }{\sigma })+\sum_{z_{t}^{*}={\text{VaR}}_{p,t}^{\alpha }}\text{ln}(1-\Phi (\frac{{\text{VaR}}_{p,t}^{\alpha }-\mu }{\sigma })) \end{array}$$ (27)

The relevant LR statistic is defined as

$$\begin{array}{c} {\text{LR}}_{\text{tail}}^{\alpha }=-2(L^{\alpha }\left(0,1\right)-L^{\alpha }(\hat{\mu },{\hat{\sigma }}^{2}))∼\chi_{2}^{2} \end{array}$$ (28)

#### Tick loss

The tick loss function (TLF) of [63] is an asymmetric loss function that penalizes observations that have a VaR exception more heavily.

$$\begin{array}{c} {\text{TLF}}_{t}^{\alpha }=(\alpha -I_{t})(r_{p,t}-{\text{VaR}}_{p,t}^{\alpha }) \end{array}$$ (29)

where *r*_*p*,*t*_ is the realized portfolio return on day *t* and *I*_*t*_ takes a value of one if exceptions occur and zero otherwise.

#### Quadratic loss

The quadratic loss function (QLF) of [64] penalizes a large loss more than a smaller loss at exceptions using a quadratic term.

$$\begin{array}{c} {\text{QLF}}_{t}^{\alpha }=\{\begin{array}{cc} 1+{\left(r_{p,t}-{\text{VaR}}_{p,t}^{\alpha }\right)}^{2}, & \text{if}\;\text{an}\;\text{exception}\;\text{occurs},\\ 0, & \text{else}. \end{array} \end{array}$$ (30)

where *r*_*p*,*t*_ is the realized portfolio return on day *t*.

#### Average VaR

The TLF and QLF examine the cost of capital deficit to cover a loss greater than the portfolio’s predicted VaR while ignoring the opportunity cost of keeping superfluous capital when the exception does not occur. Therefore, we follow [65] to assess the efficiency of a VaR model by utilizing the average predicted portfolio VaR.

$$\begin{array}{c} \bar{{\text{VaR}}_{p}^{\alpha }}=\frac{1}{T}\sum_{t=1}^{T}{\text{VaR}}_{p,t}^{\alpha } \end{array}$$ (31)

#### Standard deviation of VaR

The lower the standard deviation of predicted VaR, the less uncertain the amount of capital required to cover against unexpected portfolio losses. The final measure is the standard deviation of the portfolio predicted VaR proposed by [65].

$$\begin{array}{c} SD\left({\text{VaR}}_{p}^{\alpha }\right)=\sqrt{\frac{1}{T}\sum_{t=1}^{T}{\left({\text{VaR}}_{p,t}^{\alpha }-\bar{{\text{VaR}}_{p}^{\alpha }}\right)}^{2}} \end{array}$$ (32)

## 1 Results and discussion

### 1.1 Statistical evaluation

We first perform a statistical assessment of portfolio volatility forecast. Table 5 represents the Mincer-Zarnowitz regression results, as well as the RMSE and MAE of competing volatility models over the out-of-sample period. The assumption of innovation distribution has no substantial impact on the performance of the univariate GARCH models. The coefficient estimates of the conditional realized volatility models are closer to zero for the intercept and near unity for the slope with a higher *R*^2^ when compared to the univariate GARCH models. The non-significant intercepts suggest that there is no systematic bias in the volatility forecasts over the out-of-sample period, indicating that the models are well-calibrated and do not consistently over- or under-predict the volatility levels. It can been that the forecasts produced by intradaily volatility models are less biased, more efficient, and more informative. Moreover, their RMSE and MAE are lower than those produced by the GARCH models. Specifically, the ARFIMA model performs the best, followed by the ASYHAR and then the HAR models. Their performance, however, is generally indistinguishable. It is interesting to note that the DCC model outperforms all univariate GARCH models and EWMA with the weak-form efficient hypothesis not being rejected at the 5% significance level. However, the forecast of the DCC model is neither more informative nor more accurate than the forecasts produced by the intradaily volatility models.

**Table 5:** Volatility forecast evaluation.

	Inter	*p*-val	Slope	*p*-val	F-stat	*p*-val	*R* ^2^	RMSE	MAE
GARCH	0.090	0.112	0.894	0.000	22.944	0.000	0.711	0.490	0.293
GARCH-ST	0.095	0.095	0.887	0.000	26.448	0.000	0.710	0.492	0.293
GJRGARCH	0.100	0.008	0.887	0.000	31.143	0.000	0.747	0.461	0.273
GJRGARCH-ST	0.110	0.003	0.870	0.000	43.623	0.000	0.748	0.465	0.275
APARCH	0.079	0.066	0.921	0.000	13.945	0.000	0.752	0.451	0.267
APARCH-ST	0.092	0.033	0.901	0.000	23.117	0.000	0.752	0.454	0.268
FIGARCH	0.095	0.058	0.879	0.000	28.611	0.000	0.683	0.516	0.303
FIGARCH-ST	0.083	0.086	0.886	0.000	26.399	0.000	0.685	0.513	0.301
ARFIMA	-0.014	0.465	0.985	0.000	4.092	0.017	0.806	0.396	0.221
HAR	0.027	0.198	0.943	0.000	14.228	0.000	0.805	0.400	0.223
ASYHAR	0.015	0.476	0.959	0.000	8.832	0.000	0.819	0.383	0.215
EWMA	0.082	0.189	0.872	0.000	39.192	0.000	0.693	0.511	0.308
DCC-T	-0.025	0.692	1.036	0.000	2.339	0.097	0.715	0.479	0.278

**Notes:** The table reports volatility forecast evaluation based on the RMSE, MAE, and the Mincer-Zarnowitz regression including the intercept (Inter) and the slope (Slope) coefficient with *p*-value based on the Newey-West standard errors, the *R*^2^, and the F-statistics with associated *p* values for the test of the hypothesis *H*_0_: *α*_*i*,*j*_ = 0, *β*_*i*,*j*_ = 1. The out-of-sample evaluation period is from January 27, 2005 to December 31, 2009 (1,242 observations).

**Fig 2:**
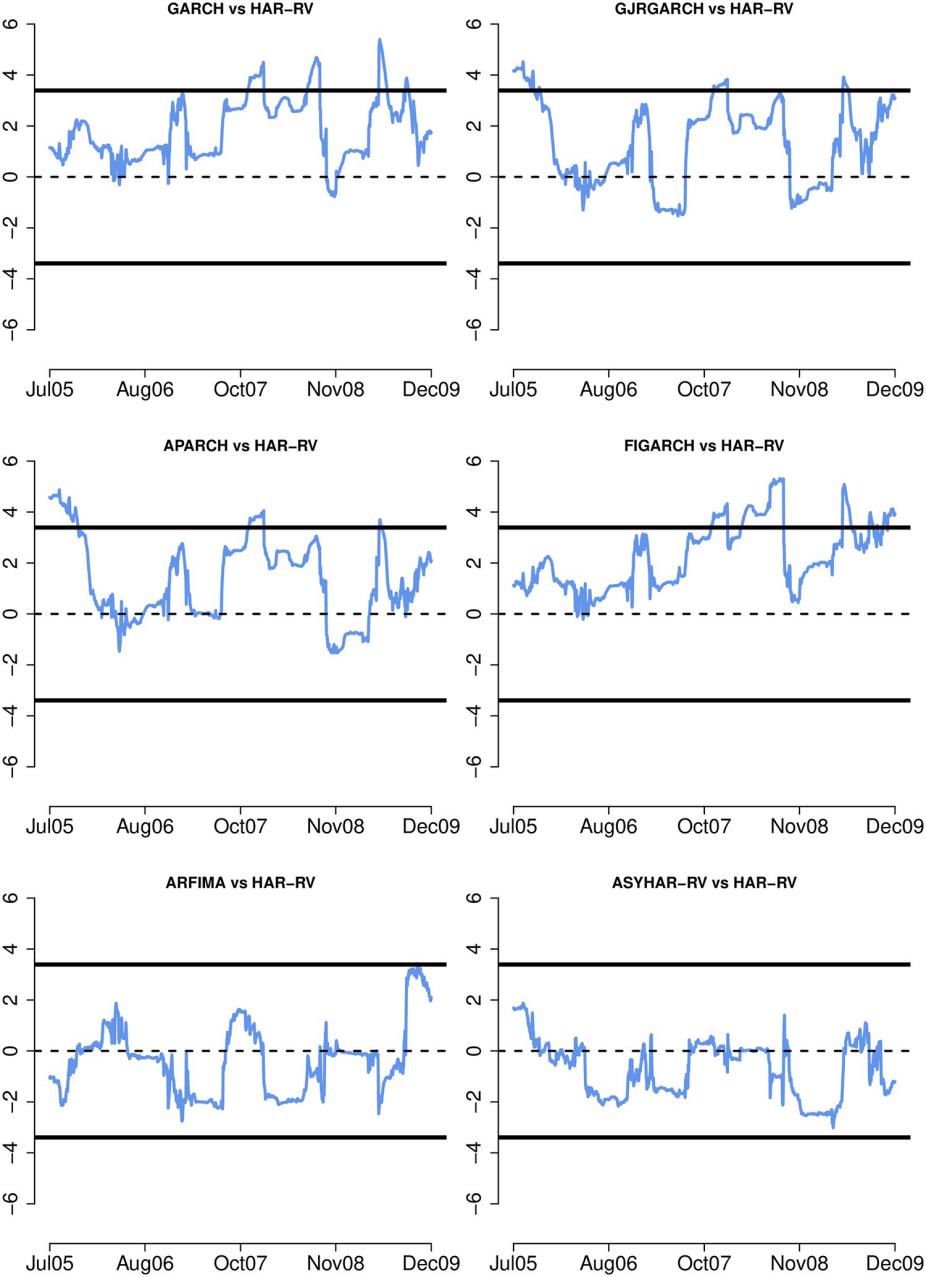
Fluctuation test I. **Notes:** The graphs show the fluctuation test statistics with the 95% critical values for the MSE differences between competing conditional volatility models. The MSE statistics are calculated using rolling windows of 124 observations over the out-of-sample evaluation period from January 27, 2005 to December 31, 2009 (1,242 observations).

**Fig 3:**
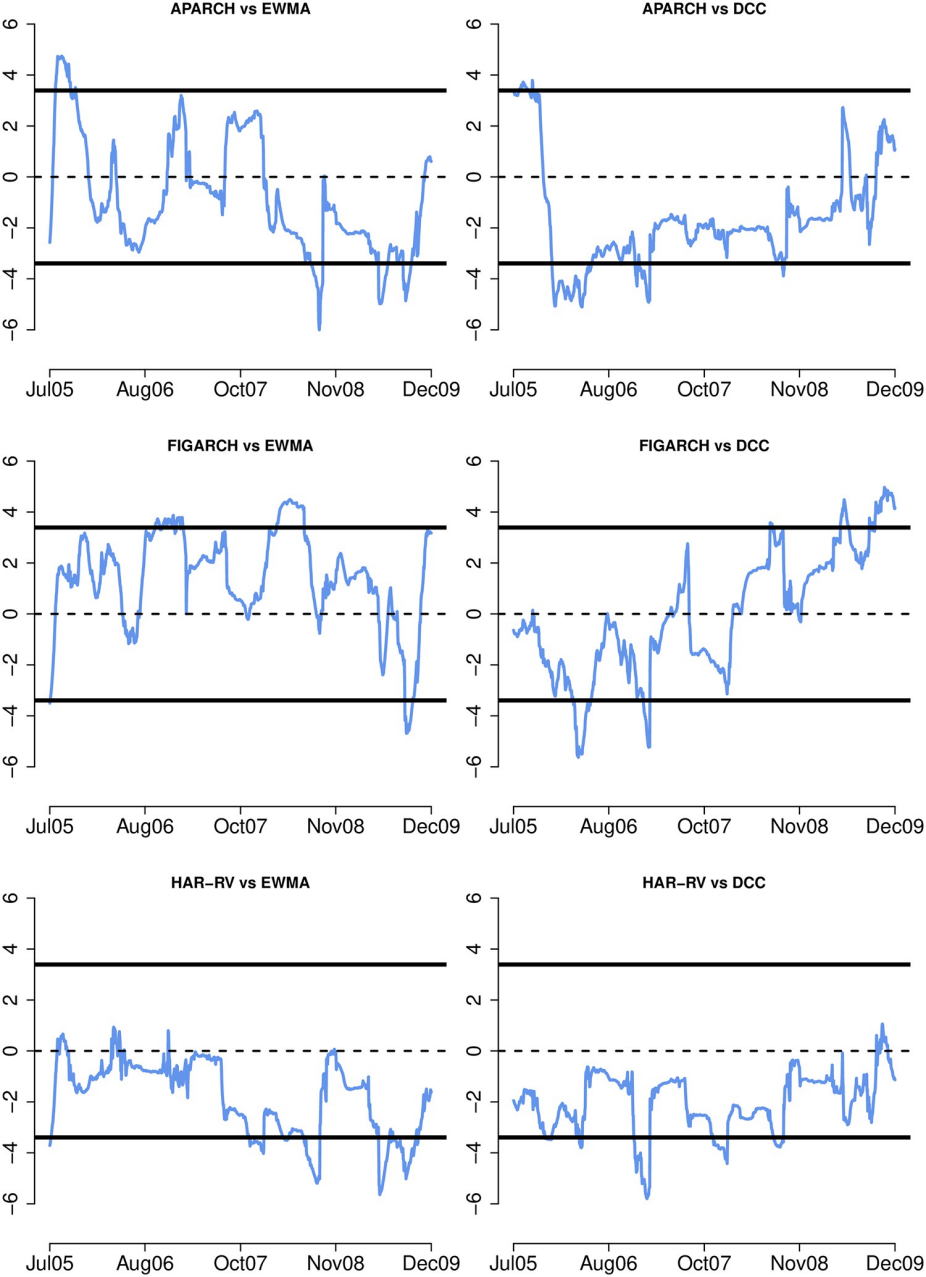
Fluctuation test II. **Notes:** The graphs show the fluctuation test statistics with the 95% critical values for the MSE differences between competing conditional volatility models. The MSE statistics are calculated using rolling windows of 124 observations over the out-of-sample evaluation period from January 27, 2005 to December 31, 2009 (1,242 observations).

Next, the fluctuation test of [66] is employed to provide insight into the models’ relative forecast accuracy over time. Fig 2 displays a sequence of differences between the MSE of the standard HAR-RV model and the univariate GARCH models with skewed student innovations over a rolling window of 124 observations (approximately 10% of the total out-of-sample observations). It also depicts the two-sided 95% critical values for testing the null hypothesis that the two models have equal out-of-sample performance at each point in time. Negative (positive) values indicate that the first model in the pair of comparisons produces better volatility forecast than the second model, i.e. the standard HAR-RV model. It is noted that all the GARCH models are less accurate than the HAR-RV model, with the upper 95% critical values being exceeded at least one point in time. The difference between ARFIMA (or ASYHAR-RV) and the standard HAR-RV is insignificant, although the ASYHAR-RV forecast appears to be more accurate. Fig 3 compares the univariate models to the multivariate EWMA and the DCC models. The relative performance of the univariate and multivariate EWMA or GARCH models varies over time, with neither consistently surpassing the other. The HAR-RV model, however, consistently delivers more accurate forecasts than the EWMA and the DCC, with the lower 95% critical values crossing frequently. Overall, the fluctuation test results show that when integrated with intraday information, the univariate models give more accurate volatility forecasts than both the univariate and multivariate GARCH models.

### 1.2 VaR evaluation

The VaR forecasts are assessed at four confidence levels *p* ∈ {0.95, 0.975, 0.99, 0.995} for both long and short trading positions. These levels have been chosen to provide a comprehensive evaluation of the models across a range of risk thresholds that are commonly used in financial risk management. Specifically, the 95% level is frequently used for standard internal risk management. It provides insight into the moderate tail risks that financial institutions might face under normal market conditions. The 99% level is a critical threshold commonly used by regulators, including the Basel Committee on Banking Supervision, for calculating market risk and determining capital requirements. It reflects significant financial stress but not the most extreme conditions. By extending the evaluation to include confidence levels of 97.5%, and 99.5%, we aim to assess the model’s performance under various extreme market scenarios and for investors with different risk appetites. This multi-threshold approach allows a more detailed and nuanced understanding of the models’ performance in predicting tail risks, which is essential for both regulatory compliance and strategic risk management.

Tables 6 and 7 show results of the unconditional and conditional coverage tests, respectively, which are qualitatively similar. First, for long positions, volatility models with normally distributed innovations fail both tests across all VaR confidence levels. This implies that the conditional normal distribution assumption is not supported by empirical evidence. When using a skewed student distribution, performance improves dramatically. For short positions, all the volatility models pass both tests with varying degrees of accuracy and the GARCH models with skewed student innovation performs better than their normally distributed counterparts at the high quantiles (i.e. 0.99 and 0.995).

**Table 6:** Unconditional coverage test.

	Long				Short			
	0.95	0.975	0.99	0.995	0.95	0.975	0.99	0.995
FHS	0.103	0.380	0.014	0.007	0.257	0.085	0.328	0.755
GARCH	0.001	0.002	0.000	0.000	0.886	0.294	0.026	0.162
GARCH-ST	0.002	0.059	0.047	0.039	0.377	0.222	0.216	0.932
GJRGARCH	0.005	0.000	0.000	0.000	0.806	0.222	0.476	0.082
GJRGARCH-ST	0.009	0.017	0.476	0.755	0.167	0.085	0.476	0.293
APARCH	0.002	0.000	0.000	0.000	0.377	0.164	0.328	0.039
APARCH-ST	0.003	0.011	0.328	0.755	0.080	0.059	0.659	0.491
FIGARCH	0.001	0.000	0.001	0.000	0.783	0.164	0.136	0.162
FIGARCH-ST	0.002	0.059	0.047	0.614	0.449	0.164	0.328	0.755
ARFIMA	0.025	0.040	0.328	0.491	0.225	0.452	0.305	0.932
ARFIMA-ST	0.080	0.482	0.305	0.614	0.907	0.848	0.305	0.932
HAR	0.080	0.027	0.082	0.162	0.501	0.452	0.305	0.614
HAR-ST	0.132	0.380	0.305	0.614	0.615	0.573	0.092	0.614
ASYHAR	0.034	0.007	0.047	0.293	0.501	0.125	0.680	0.614
ASYHAR-ST	0.080	0.222	0.305	0.932	0.806	0.452	0.475	0.614
EWMA	0.005	0.000	0.000	0.000	0.590	0.294	0.136	0.082
DCC-T	0.449	0.597	0.870	0.491	0.177	0.345	0.305	0.151

**Notes:** The table reports the *p*-values of the unconditional coverage tests of all competing models. The out-of-sample evaluation period is from January 27, 2005 to December 31, 2009 (1,242 observations). Note that a *p*-value greater than 5% suggests that we cannot reject the null hypothesis that the forecasting ability is adequate.

**Table 7:** Conditional coverage test.

	Long				Short			
	0.95	0.975	0.99	0.995	0.95	0.975	0.99	0.995
FHS	0.029	0.680	0.032	0.023	0.525	0.214	0.503	0.916
GARCH	0.000	0.005	0.000	0.000	0.805	0.573	0.058	0.346
GARCH-ST	0.003	0.156	0.100	0.106	0.607	0.468	0.367	0.968
GJRGARCH	0.001	0.001	0.000	0.001	0.955	0.143	0.646	0.201
GJRGARCH-ST	0.002	0.011	0.646	0.916	0.302	0.214	0.646	0.539
APARCH	0.000	0.001	0.000	0.000	0.607	0.371	0.503	0.106
APARCH-ST	0.000	0.007	0.503	0.916	0.148	0.156	0.773	0.749
FIGARCH	0.002	0.000	0.002	0.000	0.811	0.371	0.252	0.346
FIGARCH-ST	0.008	0.150	0.100	0.863	0.690	0.371	0.503	0.916
ARFIMA	0.005	0.026	0.503	0.749	0.297	0.413	0.553	0.968
ARFIMA-ST	0.002	0.283	0.553	0.863	0.325	0.467	0.553	0.968
HAR	0.021	0.017	0.164	0.346	0.731	0.413	0.553	0.863
HAR-ST	0.039	0.232	0.553	0.863	0.578	0.447	0.233	0.863
ASYHAR	0.008	0.004	0.100	0.539	0.731	0.200	0.832	0.863
ASYHAR-ST	0.021	0.143	0.553	0.968	0.699	0.413	0.714	0.863
EWMA	0.013	0.001	0.000	0.001	0.401	0.185	0.252	0.201
DCC-T	0.442	0.868	0.860	0.749	0.398	0.546	0.553	0.354

**Notes:** The table reports the *p*-values of the conditional coverage tests of all competing models. The out-of-sample evaluation period is from January 27, 2005 to December 31, 2009 (1,242 observations). Note that a *p*-value greater than 5% suggests that we cannot reject the null hypothesis that the forecasting ability is adequate.

If we focus on the long positions and models with non-normal distributed innovations, the DCC-T model outperforms all the univariate GARCH models since it passes both tests across all quantiles. Among the univariate GARCH models, the FHS performs relatively well at the lower quantiles, but fails at the high quantiles. The GJRGARCH-ST, APARCH-ST and FIGARCH-ST models are the opposite. This suggests that GARCH models with leverage or long memory improve VaR forecasts at high confidence levels, but are less accurate at lower confidence levels. It is worth noting that the intradaily models enhance VaR forecasts across all confidences levels when compared to the standard GARCH model. The HAR-ST model, in particular, does not reject the null hypothesis of correct unconditional and conditional coverage at the 5% significance level for all VaR quantiles.

Table 8 shows the results of the Berkowitz test for exception magnitudes which is generally consistent with the frequency tests. For example, volatility models with normally distributed innovations are rejected across all left quantiles, whereas some of them such as GARCH, GJRGARCH, and APARCH, are rejected across right quantiles. These volatility models perform better when combined with a skewed student distribution. Furthermore, the GJRGARCH and APARCH models improve standard GARCH’s accuracy at the 99% and 99.5% VaR confidence level for the long position, but remain inaccurate at lower quantiles. It is interesting to note that, despite passing the frequency tests, the DCC-T model fails the Berktowitz test at lower left quantiles. Several factors may contribute to the underperformance of the DCC-T model in this context. First, the DCC-T model’s reliance on the Student’s t-distribution assumption for the innovation distribution may not be sufficient to accurately forecast the extreme market movements, as these events often exhibit both skewness and heavy tails. Moreover, the complexity of the DCC-T model, with its additional parameters for estimating dynamic conditional correlations, poses potential estimation challenges. During periods of extreme market movements, the model may struggle to accurately update its correlation estimates, resulting in less reliable VaR forecasts at the lower quantiles where accurate prediction of extreme losses is critical. On the other hand, the intradaily models with skewed student distributions pass the tests across all left and right quantiles. Therefore, these results, when combined with the frequency tests, suggest that intradaily volatility models outperform GARCH models in terms of VaR forecast performance.

**Table 8:** Berkowitz test.

	Long				Short			
	0.95	0.975	0.99	0.995	0.95	0.975	0.99	0.995
FHS	0.151	0.108	0.045	0.052	0.156	0.108	0.123	0.141
GARCH	0.000	0.000	0.000	0.000	0.073	0.043	0.023	0.025
GARCH-ST	0.008	0.155	0.080	0.013	0.085	0.061	0.049	0.065
GJRGARCH	0.000	0.000	0.000	0.000	0.087	0.048	0.056	0.036
GJRGARCH-ST	0.033	0.051	0.770	0.915	0.141	0.095	0.127	0.108
APARCH	0.000	0.000	0.000	0.000	0.024	0.016	0.017	0.010
APARCH-ST	0.013	0.037	0.618	0.827	0.055	0.036	0.058	0.048
FIGARCH	0.000	0.000	0.000	0.000	0.235	0.140	0.124	0.110
FIGARCH-ST	0.008	0.142	0.039	0.842	0.233	0.167	0.176	0.197
ARFIMA	0.008	0.009	0.006	0.002	0.994	0.994	0.991	0.994
ARFIMA-ST	0.170	0.566	0.418	0.333	0.879	0.910	0.979	0.955
HAR	0.004	0.004	0.005	0.002	0.978	0.986	0.998	0.998
HAR-ST	0.309	0.468	0.387	0.635	0.766	0.903	0.969	0.950
ASYHAR	0.001	0.001	0.002	0.001	0.899	0.964	0.939	0.946
ASYHAR-ST	0.175	0.330	0.325	0.811	0.744	0.831	0.889	0.893
EWMA	0.000	0.000	0.000	0.000	0.422	0.309	0.272	0.245
DCC-T	0.001	0.016	0.082	0.053	0.202	0.239	0.167	0.189

**Notes:** The table reports the *p*-values of the Berkowitz test of all competing models. The out-of-sample evaluation period is from January 27, 2005 to December 31, 2009 (1,242 observations). Note that a *p*-value greater than 5% indicates that we cannot reject the null hypothesis that the forecasting ability is adequate.

To further investigate the relative performance of GARCH versus intradaily models, we now focus on the loss functions which take the exception frequency and magnitude into account simultaneously. All univariate models with normally distributed innovations are excluded from the comparison, because they are less accurate in VaR forecasts than those with skewed student distributions, particularly for the long position. The HAR-ST model is used as a benchmark for comparison since it provides adequate VaR forecasts with the simplest specification among the intradaily models. Tables 9 and 10 report [67] test statistics with *p*-values. The null hypothesis is that the mean difference between each model and the HAR-ST model in tick loss or quadratic loss function values is equal to zero. Positive (negative) statistics indicate that the corresponding models have a bigger (smaller) loss, meaning that they are less (more) accurate than the HAR-ST model. It can be seen that the statistics for all GARCH models are generally positive across all quantiles. The null hypothesis of identical tick loss values is rejected in 64% and 80% of 56 cases (across 7 GARCH models, 4 confidence levels for both long and short positions), respectively. Similarly, at the 5% and 10% significance levels, the null hypothesis of equal Lopez loss value is rejected in 56% and 70% of the cases, respectively. The ARFIMA-ST and ASYHAR-ST models contain some negative tick loss values, but they are generally insignificant at the conventional significance levels. The results suggest that intradaily volatility models are more accurate at forecasting VaR than both univariate and multivariate GARCH models. There are no statistically significant differences in performance among intradaily models.

**Table 9:** Tick loss.

	Long				Short			
	0.95	0.975	0.99	0.995	0.95	0.975	0.99	0.995
FHS	2.637	2.607	1.960	1.666	2.361	2.561	2.260	1.583
	0.008	0.009	0.050	0.096	0.018	0.011	0.024	0.114
GARCH-ST	2.743	2.427	1.964	0.928	2.392	2.749	2.377	1.737
	0.006	0.015	0.050	0.354	0.017	0.006	0.018	0.083
GJRGARCH-ST	2.533	2.933	2.503	1.555	2.680	2.492	2.470	1.766
	0.011	0.003	0.012	0.120	0.007	0.013	0.014	0.078
APARCH-ST	2.269	2.651	2.127	1.336	2.498	2.264	2.129	1.375
	0.023	0.008	0.034	0.182	0.013	0.024	0.033	0.169
FIGARCH-ST	3.150	2.457	1.945	-0.517	2.506	2.517	2.434	1.864
	0.002	0.014	0.052	0.606	0.012	0.012	0.015	0.063
ARFIMA-ST	-0.104	0.010	-1.448	-0.527	-0.093	0.786	-0.038	-1.115
	0.917	0.992	0.148	0.598	0.926	0.432	0.970	0.265
ASYHAR-ST	1.233	1.268	0.821	0.907	-0.589	-0.509	0.427	-1.746
	0.218	0.205	0.412	0.365	0.556	0.611	0.669	0.081
EWMA	2.800	2.356	1.641	1.516	2.384	2.759	2.473	1.600
	0.005	0.019	0.101	0.130	0.017	0.006	0.014	0.110
DCC-T	1.956	1.822	1.234	0.729	2.169	2.894	2.709	1.930
	0.051	0.069	0.217	0.466	0.030	0.004	0.007	0.054

**Notes:** The table reports the two-sided Diebold-Mariano test statistics with *p*-values underneath based on the mean difference of tick loss values between each model and the HAR-ST model. The out-of-sample evaluation period is from January 27, 2005 to December 31, 2009 (1,242 observations). Note that positive (negative) statistics indicate the corresponding model has a greater (lower) tick loss than the HAR-ST model.

**Table 10:** Quadratic loss.

	Long				Short			
	0.95	0.975	0.99	0.995	0.95	0.975	0.99	0.995
FHS	1.504	1.523	2.267	2.687	2.393	3.030	2.563	1.314
	0.133	0.128	0.024	0.007	0.017	0.002	0.010	0.189
GARCH-ST	2.467	1.971	2.323	2.358	2.180	2.690	2.665	1.061
	0.014	0.049	0.020	0.019	0.029	0.007	0.008	0.289
GJRGARCH-ST	2.678	2.670	2.169	1.639	2.786	3.192	2.796	1.702
	0.007	0.008	0.030	0.101	0.005	0.001	0.005	0.089
APARCH-ST	2.734	2.620	2.420	1.775	3.093	3.284	2.621	1.500
	0.006	0.009	0.016	0.076	0.002	0.001	0.009	0.134
FIGARCH-ST	2.858	2.011	2.413	-0.435	2.028	2.659	2.587	1.116
	0.004	0.045	0.016	0.664	0.043	0.008	0.010	0.265
ARFIMA-ST	1.423	0.200	0.111	0.027	-0.421	1.258	0.905	0.836
	0.155	0.842	0.912	0.978	0.674	0.208	0.366	0.403
ASYHAR-ST	1.232	1.198	1.080	1.435	-0.111	0.173	2.021	0.123
	0.218	0.231	0.280	0.152	0.911	0.863	0.043	0.902
EWMA	1.998	2.265	2.571	2.375	0.546	2.368	2.840	2.108
	0.046	0.024	0.010	0.018	0.585	0.018	0.005	0.035
DCC-T	1.168	1.135	1.262	1.151	0.066	0.990	1.220	0.217
	0.243	0.256	0.207	0.250	0.947	0.322	0.223	0.828

**Notes:** The table reports the two-sided Diebold-Mariano test statistics [67] with *p*-values underneath based on the mean difference of quadratic loss values between each model and the HAR-ST model. The out-of-sample evaluation period is from January 27, 2005 to December 31, 2009 (1,242 observations). Note that positive (negative) statistics indicate the corresponding model has a greater (lower) tick loss than the HAR-ST model.

Finally, the mean and standard deviation of VaR estimates are calculated with results reported in Table 11. For the long position, intradaily models have marginally higher average VaR values but lower standard deviation than GARCH models, but the difference is less than 1% for the mean and within 2% for the standard deviation. For the short position, the intradaily models show lower mean and smaller standard deviations across all confidence levels, with a difference of about 1% for the mean and 3% for the standard deviation. Overall, the results show that intradaily models reduce the variability of VaR estimations while improving accuracy without increasing VaR levels significantly.

**Table 11:** VaR efficiency.

	Long				Short			
	0.95	0.975	0.99	0.995	0.95	0.975	0.99	0.995
**Panel A: Average VaR**								
FHS	-1.894	-2.275	-2.802	-3.124	1.654	2.009	2.589	2.860
GARCH-ST	-1.749	-2.203	-2.819	-3.310	1.669	2.064	2.595	3.017
GJRGARCH-ST	-1.796	-2.249	-2.850	-3.319	1.652	2.027	2.523	2.907
APARCH-ST	-1.771	-2.211	-2.794	-3.246	1.629	1.996	2.478	2.849
FIGARCH-ST	-1.773	-2.235	-2.863	-3.364	1.690	2.091	2.631	3.059
ARFIMA-ST	-1.809	-2.245	-2.812	-3.246	1.668	2.036	2.511	2.871
HAR-ST	-1.820	-2.261	-2.837	-3.278	1.671	2.041	2.519	2.883
ASYHAR-ST	-1.818	-2.256	-2.824	-3.257	1.656	2.018	2.483	2.836
DCC-T	-1.848	-2.263	-2.792	-3.188	1.848	2.263	2.792	3.188
Interdaily (average)	-1.805	-2.239	-2.820	-3.258	1.690	2.075	2.601	2.980
Intradaily (average)	-1.800	-2.247	-2.837	-3.296	1.676	2.056	2.554	2.938
**Panel B: Std VaR**								
FHS	1.518	1.850	2.325	2.571	1.304	1.571	1.981	2.214
GARCH-ST	1.397	1.774	2.295	2.716	1.306	1.623	2.059	2.410
GJRGARCH-ST	1.490	1.884	2.417	2.840	1.354	1.666	2.084	2.414
APARCH-ST	1.445	1.822	2.330	2.732	1.315	1.615	2.015	2.329
FIGARCH-ST	1.373	1.738	2.239	2.641	1.292	1.602	2.023	2.359
ARFIMA-ST	1.397	1.738	2.185	2.528	1.242	1.516	1.872	2.144
HAR-ST	1.460	1.819	2.291	2.655	1.293	1.581	1.954	2.241
ASYHAR-ST	1.459	1.815	2.278	2.633	1.276	1.554	1.912	2.185
DCC-T	1.336	1.642	2.036	2.336	1.336	1.642	2.036	2.336
Interdaily (average)	1.426	1.785	2.274	2.639	1.318	1.620	2.033	2.344
Intradaily (average)	1.410	1.765	2.238	2.608	1.276	1.566	1.950	2.248

**Notes:** The table reports the mean and the standard deviation of VaR values under the competing models over the out-of-sample evaluation period from January 27, 2005 to December 31, 2009 (1,242 observations). The average across all interdaily models, i.e. GARCH and intradaily models are also reported.

### Robustness tests

There are two main limitations of the above analysis. First, the study is based on an equally weighted portfolio, however in reality portfolio managers may hold a variety of allocations based on their target returns, risk tolerance, and market view. As a result, it would be worthwhile to investigate if the outcomes are sensitive to the changes in portfolio risk profiles. Second, the out-of-sample period includes the 2008 financial crisis. [68] suggest that a Global Financial Crisis (GFC)-robust approach should not be overly sensitive to the period chosen for analysis so that risk managers do not have to continually adjust the rules for creating VaR forecasts when moving between tranquil and turbulent periods. Therefore, it would be interesting to examine whether the relative performance of each model remains stable throughout market conditions. In this part, we perform robustness tests to address the two constraints, respectively.

#### Portfolio allocations

**Fig 4:**
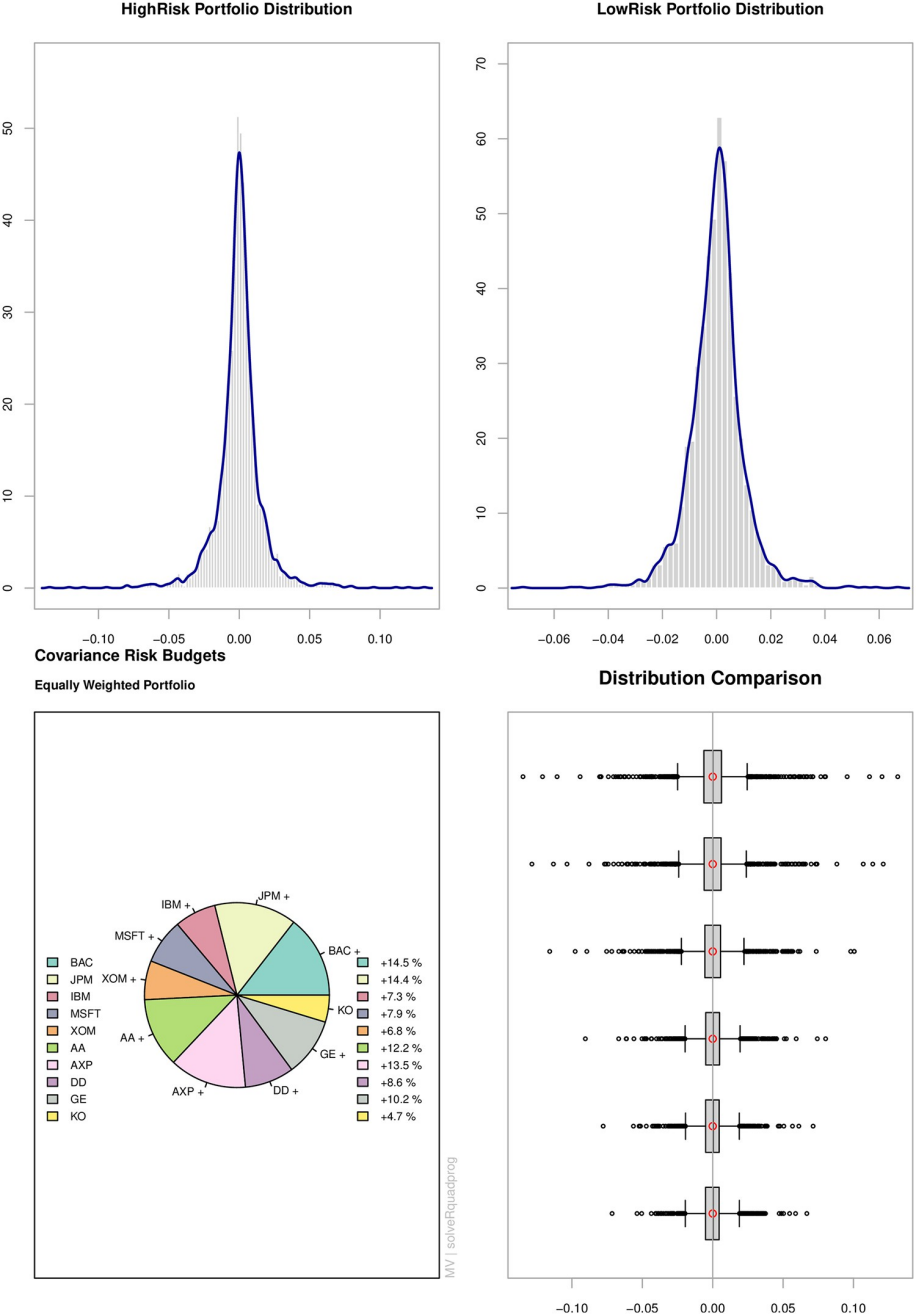
Portfolio distribution. **Notes:** The pie chart shows the covariance risk contribution of each stock in an equally-weighted portfolio. The HighRisk portfolio is made up of the top 5 risky stocks, which are equally weighted. The LowRisk portfolio consists of the remaining 5 stocks, which are also equally weighted. Their return densities are shown in the upper panel. The bottom right graph (from top to bottom) shows the return distribution of the HighRisk portfolio mixed with 0%, 10%, 30%, 70%, 90% and 100% of LowRisk portfolio.

The pie chart of Fig 4 shows the covariance risk contribution of individual stock in the equally weighted portfolio ranges from 4.7% to 14.5%. We classify the entire stock population into two groups according to their risk contribution. The first category is a HighRisk portfolio, which is composed of an equal weighting of the top five risky stocks (i.e. BAC, JPM, AXP, AA, and GE). The second group is a LowRisk portfolio comprised of the remaining stocks (i.e. IBM, MSFT, XOM, DD, and KO) that are also equally weighted. The density graphs in the upper panel show that the HighRisk portfolio is more leptokurtic than the LowRisk portfolio. In order to fully assess the impact of different risk exposures on portfolio performance, we have systematically mixed the HighRisk and LowRisk portfolios in specific proportions. The mix ratios of 90%, 70%, 30%, and 10% for the HighRisk portfolio, balanced by 10%, 30%, 70%, and 90% for the LowRisk portfolio, were chosen to represent a wide range of risk scenarios, from predominantly high-risk to predominantly low-risk exposures. This selection was strategically designed to illuminate the effects of risk concentration and diversification within a portfolio. By incrementally adjusting the proportion of HighRisk and LowRisk stocks, we can observe the corresponding changes in the overall risk profile and performance metrics of the portfolio. These specific intervals have been chosen to ensure that the transitions between different levels of risk exposure are distinct and meaningful, allowing for a clear analysis of how incremental shifts in risk allocation affect portfolio performance. The boxplot displays the changes in portfolio distribution as a result of changes in allocation between HighRisk and LowRisk groups.

**Fig 5:**
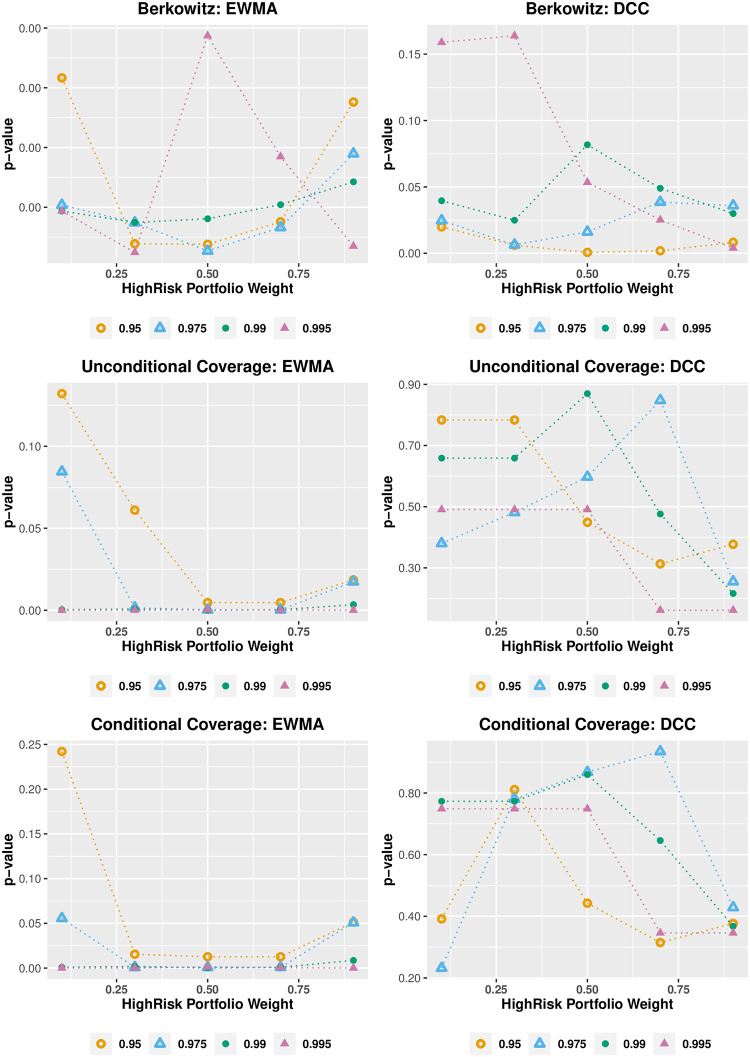
Portfolio allocation: Univariate models. **Notes:** The graphs show the *p*-values of Berkowitz, unconditional and conditional coverage tests for the VaR models (HAR-ST and GARCH-ST) under four confidence levels, i.e. 95%, 97.5%, 99%, and 99.5%. The portfolio includes long positions in 10 DJIA stocks with allocations to the HighRisk portfolio ranging from 10%, 30%, 50%, 70%, and 90% with the remainder allocated to the LowRisk portfolio. The out-of-sample evaluation period is from January 27, 2005 to December 31, 2009 (1,242 observations).

**Fig 6:**
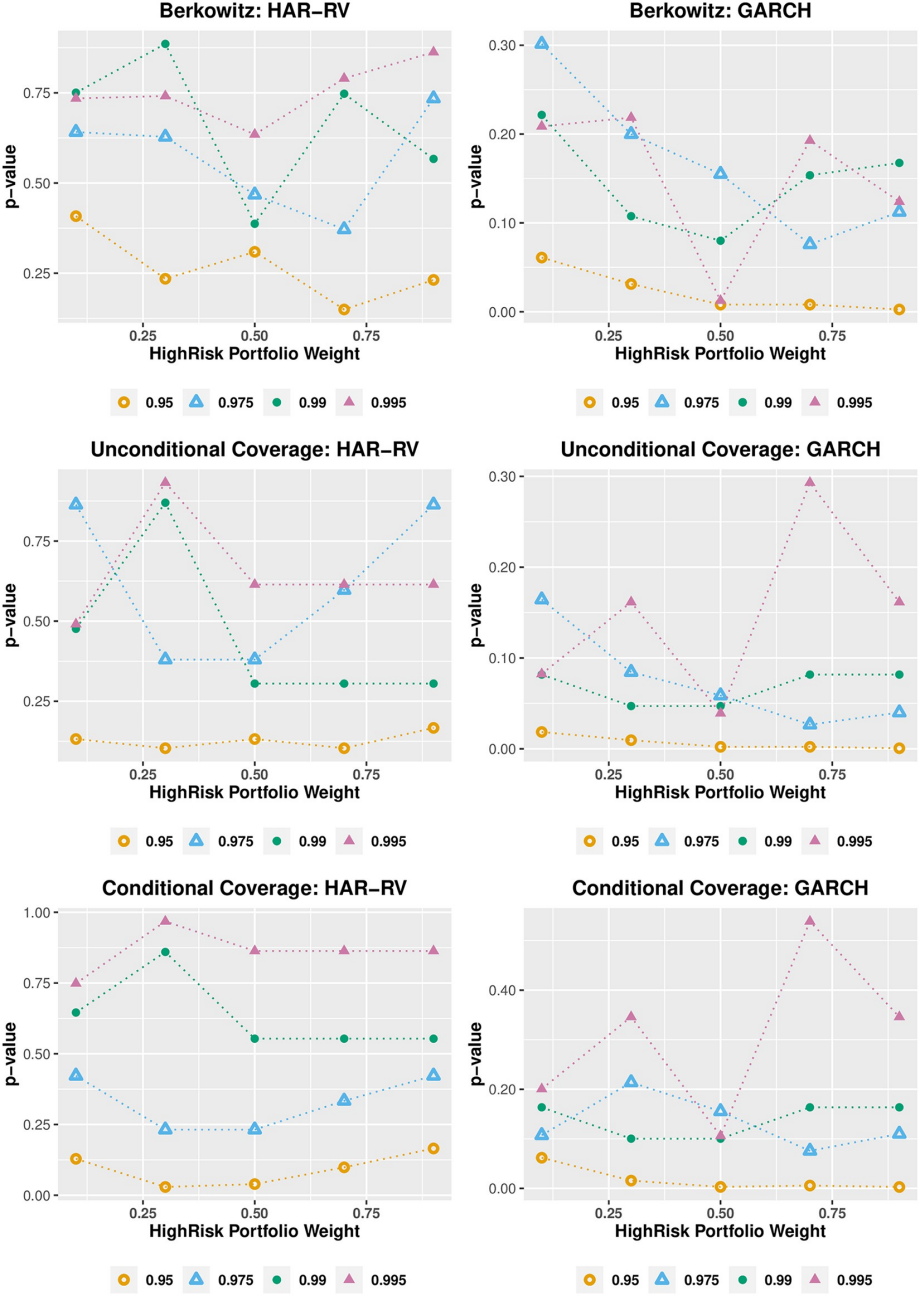
Portfolio allocation: Multivariate models. **Notes:** The graphs show the *p*-values of Berkowitz, unconditional and conditional coverage tests for the VaR models (EWMA and DCC-T) under four confidence levels, i.e. 95%, 97.5%, 99%, and 99.5%. The portfolio includes long positions in 10 DJIA stocks with allocations to the HighRisk portfolio ranging from 10%, 30%, 50%, 70%, and 90% with the remainder allocated to the LowRisk portfolio. The out-of-sample evaluation period is from January 27, 2005 to December 31, 2009 (1,242 observations).

Fig 5 compares the VaR performance of HAR-ST and GARCH-ST with varying allocation weightings to HighRisk and LowRisk portfolios. We examine representative interdaily and intradaily models rather than entire model set to reduce the amount of estimation effort for sensitivity analysis. The *p*-values of the Berkowitz, unconditional, and conditional coverage tests are presented. It is worth noting that both models are less accurate at the VaR 95% confidence level. The HAR-ST consistently outperforms the GARCH-ST across all confidence levels and portfolio risk profiles. The *p*-values of the tests fluctuate as the allocation changes. However, the results for unconditional and conditional coverage tests are fairly consistent, and there appears to be little evidence that the HAR-ST model’s performance is deteriorating for scenarios with very low or high portfolio risk. Fig 6 compares the performance of the multivariate EWMA and the DCC-T. The EWMA model fails the Berkowitz test regardless of the portfolio risk profile. Moreover, the unconditional and conditional coverage tests can only be passed for a very low risk profile at the VaR 95% confidence level. The DCC-T model generally performs poorly in the Berkowitz test and its unconditional and conditional coverage test performance deteriorates as the portfolio’s risk increases. Overall, the sensitivity analysis shows that the superior performance of HAR-ST model is robust against the changes in the portfolio’s risk profiles.

#### Sub-periods

**Fig 7:**
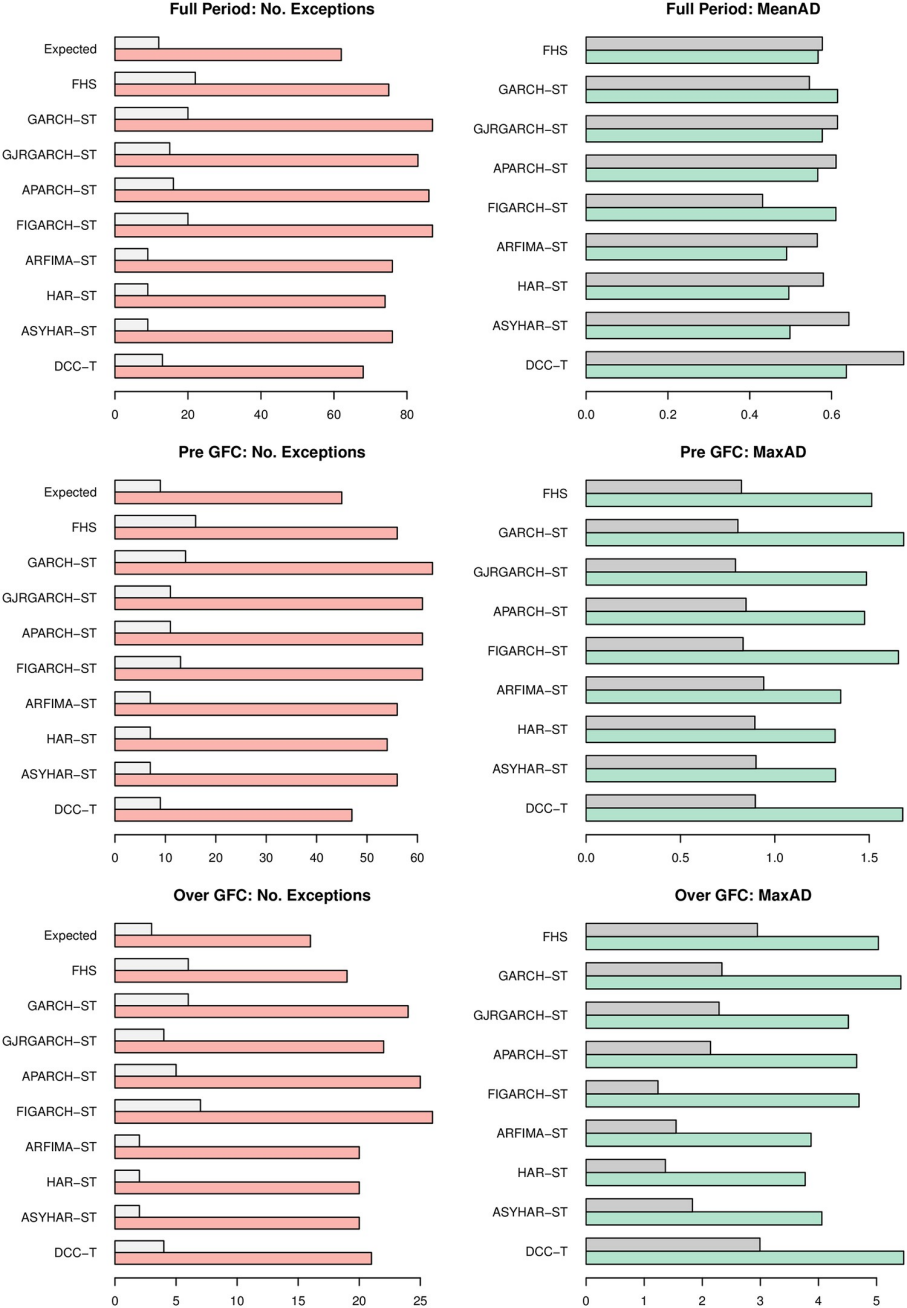
Sub-periods analysis. **Notes:** The graphs show the number of exceptions, mean absolute deviation (MeanAD), and maximum absolute deviation (MaxAD) over different sample periods. The full out-of-sample evaluation period is from January 27, 2005 to December 31, 2009. The Pre global financial crisis (GFC) is from January 27, 2005 to August 29, 2008. The over GFC period is from September 02, 2008 to December 31, 2009. Under each model, the upper bar is for VaR99 while the lower bar is for VaR95. The expected number of exceptions is shown at the top of each graph on the left.

We divide the entire out-of-sample evaluation period into two sub-periods. The pre-GFC starts from January 27, 2005 to August 29, 2008. The over-GFC period covers from September 02, 2008 to December 31, 2009. The left panel of Fig 7 displays the number of VaR backtesting exceptions before and during GFC. The expected number of exceptions is shown at the top of each graph as a reference. The right panel shows the mean absolute deviation of loss (MeanAD) throughout the full out-of-sample period and the maximum absolute deviation of loss (MaxAD) over the two sub-periods. The MeanAD (or MaxAD) measures the average (or maximum) absolute deviation of the violations from the VaR forecasts [16]. For each competing model, the upper bar is based on the results for VaR at the 99% confidence level while the lower bar is based on the 95% confidence level. The regulatory VaR backtesting is based on the comparison between one-day VaR at the 99% confidence level and the realized profit and loss [69].

It can be seen that the univariate GARCH models produce more exceptions than expected for both confidence levels while the intradaily models improve the VaR performance. The results are generally consistent before and during GFC. For example, the FHS forecast is more accurate at 95% confidence but less so at 99% confidence. The GJRGARCH and APARCH models enhance the VaR forecasts at 99% confidence to some extent, but still generate more exceptions than expected. The DCC-T model produces the number of exceptions that are closest to the expected level at the 95% confidence before the GFC, but its MeanAD is substantially larger than the other models throughout the entire period, which is consistent with the Berkowitz test result. The intradaily models appear to be conservative at the 99% confidence level. The HAR-ST model, in particular, produces the smallest MaxAD before and during GFC. Therefore, the intradaily models would be favoured by financial institutions pursuing sufficient capital or regulators seeking financial system stability.

## Discussion

This paper illuminates the advantages of leveraging intraday data for more precise portfolio variance and tail risk forecasting. Our findings are in line with recent studies that have highlighted the importance of capturing the nuances of market behaviour through high-frequency data. For instance, recent studies [70, 71] have demonstrated the effectiveness of high-frequency data in improving the accuracy of volatility forecasts for stock markets. Similarly, recent studies such as [32, 33, 72] have shown that incorporating high-frequency data can lead to more robust VaR estimates by better capturing the intraday dynamics of financial markets. However, our research extends the previous work in the following ways:

First, we critically address a gap in the existing literature by questioning the widespread belief in the superiority of multivariate GARCH models over their univariate counterparts. Previous studies, such as [5, 6], have shown that multivariate GARCH models, despite their complexity, do not significantly improve volatility forecasting. We show that univariate realized volatility models, particularly when augmented with high-frequency intraday data, not only outperform conventional multivariate models in terms of efficiency and predictive accuracy but also exhibit remarkable capability in high-dimensional settings. This challenges the notion of multivariate complexity as a proxy for predictive ability by demonstrating that intraday data mitigates the noise inherent in daily returns, which often obscures the true volatility signal [73]. The findings highlight the under-appreciated potential of univariate models as robust tools for both risk management and other applications dependent on volatility forecasting, such as derivative asset pricing.

Second, we extend the work of [20, 36] by investigating the distributions of realized volatilities and their long-memory properties. We unravel the persistence of realized volatility within equity portfolios and show that its structure can be captured well using univariate ARFIMA or HAR-RV models. It has been found that the efficacy of these models is broadly equivalent in predicting the realized volatility of portfolios, a finding consistent with [36] research within single indices. Given the striking similarities in realized volatility patterns within and across asset classes [35], the analysis could be expanded in a larger-dimensional setting or other asset classes as future research.

Third, our research represents a significant methodological breakthrough by extending the application of univariate realized volatility modelling from single assets or indices to the complex scenario of portfolio VaR forecasting. This novel analysis goes beyong previous research that has focused narrowly on volatility timing strategies within asset allocation [35, 74, 75]. By demonstrating that improved volatility risk forecasts lead to more accurate predictions of portfolio tail risks, our work identifies the crucial role of adjusting the innovation distribution to account for skewness and excess kurtosis. Significantly, we find that univariate models offer a superior balance between the accurate representation of the innovation distribution and computational efficiency compared to their multivariate counterparts.

Last but not least, our study addresses and resolves a critical issue identified by [16] regarding the tendency of both univariate and multivariate GARCH models to produce either excessive or insufficient violation counts. We show that the use of univariate models calibrated with intraday volatility data significantly refines VaR forecasting performance and overcomes the shortcomings of multivariate DCC-T models, as demonstrated by the work of [4]. While the DCC-T model may be suitable for diversified portfolios in general, our analysis reveals that it does not fully capture the nuanced characteristics of riskier asset profiles within our specific dataset. This limitation leads to less accurate predictions and potentially excessive loss magnitudes in outlier scenarios for the DCC-T model. In contrast, the HAR-RV model proves to be a robust tool that effectively maintains its predictive integrity across different portfolio risk profiles, providing a versatile and user-friendly approach to reliable portfolio VaR forecasting.

## Conclusion

Our study advances the understanding of volatility forecasting by rigorously examining the efficacy of a univariate model based on high-frequency intraday data in improving portfolio VaR predictions, compared to traditional univariate and multivariate models using daily data. While previous research has applied high-frequency-based univariate models to index portfolios, our work provides a novel assessment of their robustness across diversified risk profiles and market conditions, particularly during financial crises.

We find that incorporating the skewness and excess kurtosis of return innovations into volatility models is crucial for accurate VaR forecasting. Our findings indicate that conditional realized volatility models based on high-frequency data, outperform both univariate and multivariate GARCH models. This superiority in forecasting accuracy and efficiency can be attributed to the high-frequency models’ ability to capture the fine-grained dynamics of market movements, which traditional GARCH models, relying on lower-frequency data, might miss. Moreover, we discover negligible performance differences between various conditional realized volatility models, indicating the efficacy of the standard HAR-RV model in capturing the long memory characteristic of realized volatility for portfolio VaR forecasting. The multivariate DCC model with Student *t* innovation, while effective in modeling time-varying correlations and outperforming the simpler EWMA technique, exhibits certain limitations. In particular, its sensitivity to changes in portfolio risk profiles and the magnitude of potential losses pose challenges. This may be due to its assumption of a symmetric innovation distribution, which does not always match the actual distribution of asset returns. The complexity of the model, involving the estimation of multiple parameters, also increases the risk of overfitting.

However, our approach is not without its limitations. The focus on the HAR model and its asymmetric extension was intentional, aiming to provide a focused examination of the impact of high-frequency data on volatility estimation. However, the potential of high-frequency intraday data to improve portfolio tail risk forecasting has not been fully realized and warrants further exploration. Future study could explore alternative models, such as Bayesian random fluctuation models to further validate the application of high-frequency data in risk management. Furthermore, future research could benefit from combining cutting-edge methodologies with high-frequency data. For instance, machine learning algorithms could be applied to analyze high-frequency data, while alternative data sources could be incorporated to provide a more comprehensive picture of market circumstances. The inclusion of comprehensive data indicators, distribution characteristics, and graphical analyses could further validate the application of these advanced methods in capturing market volatility and tail risks. Additionally, our empirical analysis, based on data from 2001 to 2009, captures a period of market stability and volatility, including the 2008 financial crisis. The evolving landscape of financial markets post-2009, with new regulatory frameworks and market dynamics, may influence the generalizability of our findings, suggesting the need for future research to assess the applicability of our results in the contemporary market environment. Moreover, the evaluation of Expected Shortfall (ES) forecasts, a complementary measure to VaR, would be a valuable addition to future work, providing a more comprehensive assessment of the predictive capabilities of models under different risk scenarios.

In conclusion, our research underscores the value of high-frequency intraday data in refining volatility predictions and portfolio VaR forecasts, while also highlighting areas for further investigation to enhance the practical application of risk management strategies in the financial industry.

## Supporting information

S1 Checklist Human participants research checklist. (DOCX)
